# Complete Sequencing of Five Araliaceae Chloroplast Genomes and the Phylogenetic Implications

**DOI:** 10.1371/journal.pone.0078568

**Published:** 2013-10-18

**Authors:** Rong Li, Peng-Fei Ma, Jun Wen, Ting-Shuang Yi

**Affiliations:** 1 Key Laboratory of Biodiversity and Biogeography, Kunming Institute of Botany, Chinese Academy of Sciences, Kunming, Yunnan, People’s Republic of China; 2 Plant Germplasm and Genomics Center, Germplasm Bank of Wild Species, Kunming Institute of Botany, Chinese Academy of Sciences, Kunming, Yunnan, People’s Republic of China; 3 Department of Botany, National Museum of Natural History, Smithsonian Institution, Washington, District of Columbia, United States of America; University of Vermont, United States of America

## Abstract

**Background:**

The ginseng family (Araliaceae) includes a number of economically important plant species. Previously phylogenetic studies circumscribed three major clades within the core ginseng plant family, yet the internal relationships of each major group have been poorly resolved perhaps due to rapid radiation of these lineages. Recent studies have shown that phyogenomics based on chloroplast genomes provides a viable way to resolve complex relationships.

**Methodology/Principal Findings:**

We report the complete nucleotide sequences of five Araliaceae chloroplast genomes using next-generation sequencing technology. The five chloroplast genomes are 156,333–156,459 bp in length including a pair of inverted repeats (25,551–26,108 bp) separated by the large single-copy (86,028–86,566 bp) and small single-copy (18,021–19,117 bp) regions. Each chloroplast genome contains the same 114 unique genes consisting of 30 transfer RNA genes, four ribosomal RNA genes, and 80 protein coding genes. Gene size, content, and order, AT content, and IR/SC boundary structure are similar among all Araliaceae chloroplast genomes. A total of 140 repeats were identified in the five chloroplast genomes with palindromic repeat as the most common type. Phylogenomic analyses using parsimony, likelihood, and Bayesian inference based on the complete chloroplast genomes strongly supported the monophyly of the Asian Palmate group and the *Aralia*-*Panax* group. Furthermore, the relationships among the sampled taxa within the Asian Palmate group were well resolved. Twenty-six DNA markers with the percentage of variable sites higher than 5% were identified, which may be useful for phylogenetic studies of Araliaceae.

**Conclusion:**

The chloroplast genomes of Araliaceae are highly conserved in all aspects of genome features. The large-scale phylogenomic data based on the complete chloroplast DNA sequences is shown to be effective for the phylogenetic reconstruction of Araliaceae.

## Introduction

Araliaceae (the ginseng family) consist of approximately 45 genera and 1,500 species with a wide distribution in tropical and subtropical Asia, the Pacific and Indian Ocean basins, and the Neotropics, with a few well-known genera from the north and south temperate zones [1–3]. Members of Araliaceae are characterized by mostly woody habit and often 5-merous flowers, inflorescences commonly a compound umbel, and fruit a berry mostly with 2-5 (rarely to many) seeds, yet highly variable leaf morphology (simple, palmately compound, to variously pinnately compound) [1,4,5]. The family includes a number of important medicinal plants, such as *Panax* L. (ginseng) and *Eleutherococcus* Maxim. (Siberian ginseng), and several well-known ornamentals, including *Hedera* L. (English ivy), *Schefflera* J. R. Forst. & G. Forst. (the umbrella trees), *Fatsia* Decne. & Planch., and *Polyscias* J. R. Forst. & G. Forst [1]. Many species are used for timber (e.g., *Kalopanax* Miq.) and as vegetables (e.g., *Aralia* L., *Eleutherococcus*, *Metapanax* J. Wen & Frodin, and *Oplopanax* (Torr. & Gray) Miquel) in China [6].

Previous phylogenetic studies based on sequence data from nuclear ribosomal DNA [1,2,7] and chloroplast DNA [3] have provided important insights into the evolution and diversification of Araliaceae and circumscribed three major monophyletic groups within the core Araliaceae: the Asian Palmate group, the *Polyscias*-*Pseudopanax* group, and the *Aralia*-*Panax* group. However, the phylogenetic relationships among the genera within each major group have been poorly resolved, possibly due to their rapid divergence in the early evolutionary history [1,3]. Thus, it is necessary to construct a robust phylogenetic tree to facilitate a better understanding of the intergeneric relationships and evolutionary diversification in the family.

Chloroplasts are multifunctional organelles, which possess their own genetic material, and are supposed to have originated from ancient endosymbiotic cyanobacteria [8–10]. The size of chloroplast genome in angiosperms is usually between 115 and 165 kb [11]. Typically the circular quadripartite genome consists of two inverted repeats (IRs) separated by two regions of unique DNA, the large (LSC) and small (SSC) single-copy regions [11,12]. The lack of recombination, low rates of nucleotide substitutions, and usually uniparental inheritance make plant chloroplast DNA valuable sources of genetic markers for phylogenetic analyses [9,13–15]. For instance, recent phylogenetic analyses of chloroplast genome sequences have helped confirm deep-level phylogenetic relationships derived from non-genome wide data and have revealed new relationships at various taxonomic levels, which included a well-supported sister relationship of the monocot clade to the eudicot clade [16–25].

With the emergence of next-generation sequencing, new approaches for genome sequencing have been gradually proposed due to their high-throughput, time-saving, and low-cost advantages [26]. Since the first determination of the angiosperm complete chloroplast genome of tobacco (*Nicotiana tabacum* L.) [27], the number of completely sequenced chloroplast genomes is growing rapidly. By June 2013, more than 300 complete chloroplast genomes representing the major lineages of green plants are available in the GenBank Organelle Genome Resources. It is now much more convenient to obtain chloroplast genome sequences and extend gene-based phylogenetics to phylogenomics.

At present, only two chloroplast genomes have been sequenced in the Araliaceae, including *Panax ginseng* C. A. Meyer (published as *P. schinseng* Nees) [28] and *Eleutherococcus senticosus* (Rupr. & Maxim.) Maxim [29]., which belong to two major clades (the *Aralia*-*Panax* group and the Asian Palmate group) in the family. To better understand evolution of chloroplast genome and especially to explore the potential of phylogenomics to resolve the phylogenetic relationships within Araliaceae and the close relatives, we completed chloroplast genomes of five representative species (*Aralia undulata* Hand.-Mazz., *Brassaiopsis hainla* (Buch.-Ham.) Seem., *Kalopanax septemlobus* (Thunb.) Koidz., *Metapanax delavayi* (Franch.) J. Wen & Frodin, and *Schefflera delavayi* (Franch.) Harms) using next-generation Illumina sequencing-by-synthesis technology [26]. Previous phylogenetic studies [1–3] placed the five species in two major lineages in Araliaceae, with *Aralia undulata* in the *Aralia*-*Panax* group, and the other species in the Asian Palmate group. Our goals of this study are to (1) gain insights into the evolutionary patterns of chloroplast genome in Araliaceae; and (2) explore the efficiency of chloroplast phylogenomic data in Araliaceae and evaluate the phylogenetic relationships in the Asian Palmate group from a phylogenomic perspective.

## Materials and Methods

### Ethics statement

The five sampled species were grown in Kunming Botanical Garden of the Kunming Institute of Botany, Chinese Academy of Sciences. The voucher specimens were deposited at the Herbarium of Kunming Institute of Botany (KUN), Chinese Academy of Sciences and the collectors and numbers are R. Li 551 for *Aralia undulata*, R. Li 550 for *Brassaiopsis hainla*, R. Li 552 for *Kalopanax septemlobus*, R. Li 549 for *Metapanax delavayi*, and R. Li 548 for *Schefflera delavayi*. A sample collection permit was obtained from Kunming Botanical Garden (permit number: 2011-86).

### DNA sequencing, genome assembly, and validation

We collected 50–100 g of fresh leaves from each species for chloroplast DNA isolation using an improved extraction method that included high ionic strength buffer with low pH (3.60) [11]. We used 5 μg of purified DNA for fragmentation by nebulization with compressed nitrogen, and constructed short-insert (500 bp) libraries following the manufacturer’s protocol (Illumina HiSeq 2000). DNA from the different species was indexed by tags and pooled together in one lane of an Illumina HiSeq 2000 system for sequencing at Beijing Genomics Institute (BGI) in Shenzhen, China.

The raw sequence reads included non-chloroplast DNA. To determine the accuracy of chloroplast DNA, we mapped sequence reads to the *Panax ginseng* chloroplast genome (i.e., used it as a reference genome) using Bowtie with paired-end alignment and a maximum of 3 mismatches (-v=3) [30]. The clean sequence reads were deposited in GenBank and can be accessed in the Sequence Read Archive (*Aralia undulata*: SRS455327; *Brassaiopsis hainla*: SRS455328; *Kalopanax septemlobus*: SRS455329; *Metapanax delavayi*: SRS455330; and *Schefflera delavayi*: SRS455331). Subsequently, the chloroplast genome was assembled following the method of Cronn et al. [31] with some modifications. First, we assembled raw sequence reads into contigs using SOAPdenovo [32] with an overlapping length of 31 bp. Second, contigs with a minimum length of 100 bp were aligned to the reference genome using the BLAST program, and aligned contigs were ordered according to the reference genome. Third, gaps between the *de novo* contigs were filled via direct sequencing of PCR products amplified using primers that were complementary to the end sequences of each contig (Table S1).

The four junctions between the single-copy segments and inverted repeats were validated by using PCR-based sequencing in each chloroplast genome. The primer pairs were designed based on the reference genome (Table S1). PCR products were sequenced using standard Sanger protocols on ABI 3730 xl instruments. Sanger sequences and assembled genomes were aligned using MEGA 5.0 [33] to determine if there were any differences.

### Genome annotation and whole genome comparison

The chloroplast genomes were annotated by using the program DOGMA (Dual Organellar GenoMe Annotator) [34], coupled with manual corrections for start and stop codons. Protein-coding genes were identified by using the plastid/bacterial genetic code. Intron/exon positions were determined following Sugita and Sugiura [35] with those of the *Panax ginseng* chloroplast genome as the reference [28]. We also used the program tRNAscan-SE [36] with default settings to corroborate tRNA boundaries identified by DOGMA. The program OGDRAW (OrganellarGenomeDRAW) [37] was applied to convert genetic information annotated in GenBank files into graphical maps.

To compare the overall similarities among different chloroplast genomes in Araliaceae, we obtained *Panax ginseng* (AY582139) and *Eleutherococcus senticosus* (JN637765) chloroplast genome sequences from GenBank. Pairwise alignments among seven Araliaceae chloroplast genomes were performed by the mVISTA program in LAGAN mode [38] using the annotation of *Panax ginseng* as the reference. The genetic divergence represented by *p*-distance was calculated by MEGA 5.0 [33] with the species of the Asian Palmate group as one group and those of the *Aralia*-*Panax* group as another.

### Examination of repeat structure

The program REPuter [39] was used to assess the number and location of repeats within the five Araliaceae chloroplast genomes. Following the method of Zhang et al. [24], the repeats were divided into three types: tandem, dispersed, and palindromic. For all the repeat types, constraint set in REPuter was 90% or greater sequence identity with hamming distance equal to 3. The minimum repeat size investigated was 15 bp for tandem, 30 bp for dispersed and 20 bp for palindromic, respectively. Gap size between palindromic repeats was restricted to a maximal length of 3 kb. Overlapping repeats were merged into one repeat motif whenever possible. A given region in the genome was designated as only one repeat type, and the tandem repeat was prior to dispersed repeat if one repeat motif could be identified as both tandem and dispersed repeats. After program run, tandem repeats with less than 15 bp in length and the redundant output of REPuter were manually filtered.

### Phylogenomic analyses

The seven Araliaceae chloroplast genome sequences were used for phylogenetic analysis (Table 1). Because of the close relationship of Apiaceae and Araliaceae [1,40–42], *Daucus carota* L. (DQ898156) [42] of Apiaceae was included as the outgroup (Table 1). Sequences were aligned using the program MAFFT version 5.0 [43] and edited manually. The unambiguously aligned DNA sequences were used for phylogenetic tree construction. In order to examine the phylogenetic utility of different regions, phylogenetic analyses were performed based on the following data set: (1) the complete chloroplast DNA sequences; (2) the large single-copy region; (3) the small single-copy region; (4) the inverted repeat region; (5) a set of 80 common protein coding genes; (6) the intergenic spacers region; and (7) the introns. Maximum parsimony (MP) analyses was conducted using PAUP* version 4.0b10 [44]. The most parsimonious trees were obtained with heuristic searches of 1,000 replicates with random stepwise sequence addition, tree bisection-reconnection (TBR) branch swapping, collapse of zero-length branches, multiple tree option in effect. Parsimony bootstrap values (PB) were calculated with 500 bootstrap replicates with TBR branch swapping. Maximum likelihood (ML) analyses were performed using RAxML version 7.2.6 [45]. RAxML searches relied on the general time reversible (GTR) model of nucleotide substitution with the gamma model of rate heterogeneity. The likelihood bootstrap probability (LB) of each branch was calculated in the “fast bootstrap” algorithm of RAxML used 1,000 replicates. Modeltest version 3.7 [46] was used to determine the optimal model of molecular evolution and gamma rate heterogeneity using the Akaike Information Criterion (AIC) [47]. Bayesian inference (BI) was implemented in MrBayes version 3.1.2 [48] with the model estimated above. The Markov chain Monte Carlo (MCMC) algorithm was run for 2,000,000 generations with one cold and three heated chains, starting from random trees and sampling one out of every 100 generations. The first 25% of trees were discarded as burn-in, and the remaining trees were imported into PAUP and a 50% majority-rule consensus tree was produced to obtain posterior probabilities (PP) of the clades. In all analyses, gaps introduced by the alignment were excluded.

**Table 1:** Taxa included in phylogenomic analyses of the Araliaceae.

**Classification**	**Taxon**	**GenBank**	**References**
The Asian Palmate group			
	*Brassaiopsis hainla*	KC456164	Current study
	*Eleutherococcus senticosus*	JN637765	Yi et al., 2012 [29]
	*Kalopanax septemlobus*	KC456167	Current study
	*Metapanax delavayi*	KC456165	Current study
	*Schefflera delavayi*	KC456166	Current study
The *Aralia*-*Panax* group			
	*Aralia undulata*	KC456163	Current study
	*Panax ginseng*	AY582139	Kim and Lee, 2004 [28]
Outgroup			
	*Daucus carota*	DQ898156	Ruhlman et al., 2006 [42]

Indels in exons were coded as binary characters manually in a separated matrix. Maximum parsimony (MP) analyses of this matrix was performed using PAUP* version 4.0b10 [44] to implement exhaustive tree searches. Parsimony bootstrap values (PB) were conducted under 500 replicates with TBR branch swapping.

### Genome evolutionary analyses and molecular marker identification

To examine if the different chloroplast genome regions evolved following a unique pattern in each group within Araliaceae, both the coding and noncoding regions longer than 200 bp were compared among taxa from the Asian Palmate group and the *Aralia*-*Panax* group. For each group, homologous regions of chloroplast genomes were aligned using MEGA 5.0 [33] and adjusted manually where necessary. Subsequently, the percentage of variable characters for each region in each group was calculated. Because the aim was to determine whether the evolutionary pattern of each region was distinct in each group, only numbers of nucleotide substitutions were considered.

Seven Araliaceae chloroplast genomes were used to identify rapidly evolving molecular markers which may be used for phylogenetic studies of Araliaceae at different levels. As the inverted repeat regions (IRs) accumulate point mutations more slowly than the single-copy regions (LSC and SSC) [11], only fragments from single-copy regions were considered. Molecular fragments of coding regions and noncoding regions longer than 350 bp were aligned using MEGA 5.0 [33], respectively. Then, the proportion of mutational events for each coding and noncoding region was calculated following the modified version of the formula used in Gielly and Taberlet [49]. The proportion of mutation events = (NS / L) × 100, where NS = the number of nucleotide substitutions, L = the aligned sequence length. As parsimony informative sites (PIS) are commonly used in phylogenetic analyses, the proportion of parsimony informative sites was calculated as well.

To examine phylogenetic applications of rapidly evolving molecular markers, the maximum parsimony method was used to construct the phylogenetic trees with PAUP* version 4.0b10 [44] for each marker. Heuristic tree searches were conducted with 1,000 replicates with random stepwise sequence addition, tree bisection-reconnection (TBR) branch swapping. Parsimony bootstrap values (PB) were calculated using 500 replicates with TBR branch swapping.

## Results and Discussion

### Genome assembly and validation

Each sample was sequenced with the Illumina HiSeq 2000 system, and over 200 Mb data of paired-end reads were generated. After screening these paired-end reads through alignment with reference chloroplast genomes with Bowtie software, 121,793 to 141,941 reads were mapped to the reference genome, reaching mean over 100 × coverage over the chloroplast genome. After *de novo* and reference-guided assembly, two complete chloroplast genomes were obtained. The other three chloroplast genomes had one to four gaps, which were then finished by PCR-based sequencing (Table S1).

Four junction regions between IRs and SSC/LSC in each chloroplast genome were confirmed by PCR amplifications and Sanger sequencing using primers (Table S1) designed on the basis of the reference genome. The amplified sequences from five species amounted to 21,080 bp (4,430 bp for J_LB_, 3,676 bp for J_LA_, 6,737 bp for J_SB_, and 6,237 bp for J_SA_). At the same time, we compared these sequences directly to the assembled genomes, observing no nucleotide mismatches or indels. This result also validated the accuracy of our genome sequencing and assembly.

### Genome size, content, and organization

**Figure 1:**
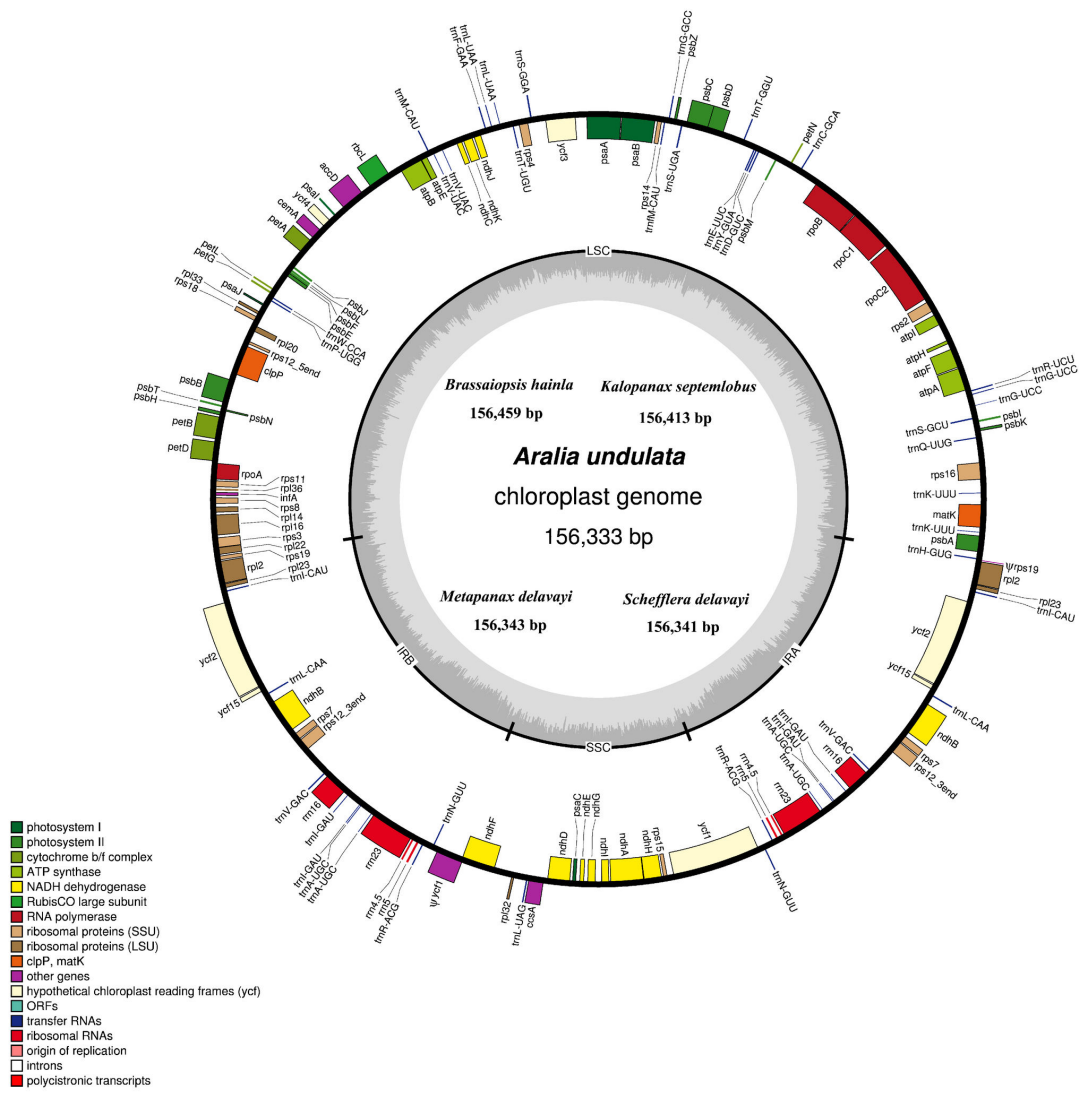
Gene map of the five Araliaceae chloroplast genomes. Genes on the outside of the outer circle are transcribed in the clockwise direction and genes on the inside of the outer circle are transcribed in the counterclockwise direction. Functional categories of genes are color-coded. Dashed area in the inner circle indicates the GC content of the chloroplast genome.

The complete nucleotide sequences of five Araliaceae chloroplast genomes range from 156,333 bp in *Aralia undulata* to 156,459 bp in *Brassaiopsis hainla*, which have been submitted to GenBank (*Aralia undulata*: KC456163; *Brassaiopsis hainla*: KC456164; *Kalopanax septemlobus*: KC456167; *Metapanax delavayi*: KC456165; *Schefflera delavayi*: KC456166). All five chloroplast genomes show a typical quadripartite structure, including a pair of IRs (25,551–26,108 bp) separated by the LSC (86,028–86,566 bp) and SSC (18,021–19,117 bp) regions (Table 2). They contain 114 unique genes consisting of 30 transfer RNA genes, four ribosomal RNA genes, and 80 protein coding genes (Table 3). In addition, there are 18 genes duplicated in the inverted repeat, making a total of 132 genes present in the five Araliaceae chloroplast genomes (Figure 1). Eighteen genes contain introns, 16 of which contain a single intron while two (*clpP* and *ycf3*) have two introns (Table 3). The chloroplast genomes consist of 50.1% to 50.3% coding regions, and the overall AT content is 61.9% to 62.2% (Table 2). The AT content of the IR regions is 56.8% to 57%, whereas, the AT contents in the LSC and SSC are 63.7% to 64% and 67.9% to 68.3%, respectively. The lower AT contents of the IR regions is largely attributed to the lower AT contents in the four rRNA (45%) genes in this region. Overall all these five chloroplast genomes are highly conserved in each aspect of genome features, such as gene size (length), gene content, gene order, and AT content.

**Table 2:** Summary of the chloroplast genome features.

	***Aralia undulata***	***Brassaiopsis hainla***	***Kalopanax septemlobus***	***Metapanax delavayi***	***Schefflera delavayi***
Size (bp)	156,333	156,459	156,413	156,343	156,341
LSC length (bp)	86,028	86,566	86,466	86,360	86,122
SSC length (bp)	18,089	18,021	18,119	18,131	19,117
IR length (bp)	26,108	25,936	25,914	25,926	25,551
Number of genes	114	114	114	114	114
Protein-coding genes	80	80	80	80	80
Structure RNAs	34	34	34	34	34
AT content (%)	61.9	62	62	62	62.2
Coding regions (%)	50.3	50.1	50.1	50.2	50.3

**Table 3:** Genes contained in chloroplast genomes (114 genes in total).

**Category**	**Group of gene**	**Name of gene**
Self replication	Ribosomal RNA genes	*rrn4.5*(×2), *rrn5*(×2), *rrn16*(×2), *rrn23*(×2)
	Transfer RNA genes	*trnA-UGC* ^*^(x2), *trnC-GCA*, *trnD-GUC*, *trnE-UUC*, *trnF-GAA*, *trnG-GCC*, *trnG-UCC* ^*^, *trnH-GUG*, *trnI-CAU*(x2), *trnI-GAU* ^*^(x2), *trnK-UUU* ^*^, *trnL-CAA*(x2), *trnL-UAA* ^*^, *trnL-UAG*, *trnfM-CAU*, *trnM-CAU*, *trnN-GUU*(x2), *trnP-UGG*, *trnQ-UUG*, *trnR-ACG*(x2), *trnR-UCU*, *trnS-GCU*, *trnS-GGA*, *trnS-UGA*, *trnT-GGU*, *trnT-UGU*, *trnV-GAC*(x2), *trnV-UAC* ^*^, *trnW-CCA*, *trnY-GUA*
	Small subunit of ribosome	*rps2*, *rps3*, *rps4*, *rps7*(x2), *rps8*, *rps11*, *rps12* ^*^(x2, part), *rps14*, *rps15*, *rps16* ^*^, *rps18*, *rps19*(x2, part)
	Large subunit of ribosome	*rpl2* ^*^(x2), *rpl14*, *rpl16* ^*^, *rpl20*, *rpl22*, *rpl23*(x2), *rpl32*, *rpl33*, *rpl36*
	RNA polymerase subunits	*rpoA*, *rpoB*, *rpoC1* ^*^, *rpoC2*
Photosynthesis	NADH dehydrogenase	*ndhA* ^*^, *ndhB* ^*^(x2), *ndhC*, *ndhD*, *ndhE*, *ndhF*, *ndhG*, *ndhH*, *ndhI*, *ndhJ*, *ndhK*
	Photosystem I	*psaA*, *psaB*, *psaC*, *psaI*, *psaJ*, *ycf3* ^**^
	Photosystem II	*psbA*, *psbB*, *psbC*, *psbD*, *psbE*, *psbF*, *psbH*, *psbI*, *psbJ*, *psbK*, *psbL*, *psbM*, *psbN*, *psbT*, *psbZ*
	Cytochrome b/f complex	*petA*, *petB* ^*^, *petD* ^*^, *petG*, *petL*, *petN*
	ATP synthase	*atpA*, *atpB*, *atpE*, *atpF* ^*^, *atpH*, *atpI*
	Large subunit of rubisco	*rbcL*
Other genes	Translational initiation factor	*infA*
	Maturase	*matK*
	Protease	clpP^**^
	Envelope membrane protein	*cemA*
	Subunit of acetyl-CoA-carboxylase	*accD*
	c-type cytochrome synthesis gene	*ccsA*
Unknown function	Conserved open reading frames	*ycf1*(x2, part), *ycf2*(x2), *ycf4*, *ycf15*(x2)

Note: one and two asterisks indicate one- and two-intron containing genes, respectively. Genes located in the IR region are indicated by (×2) after the gene name.

The structure of the five chloroplast genomes is also very similar to that of the two other reported Araliaceae chloroplast genomes [28,29]. For example, the *rps12* gene is a trans-spliced gene that the 5’ end exon is located in the LSC region and the two remaining exons are located in the IR regions (Figure 1). The *trnK-UUU* gene has the largest intron (2,513–2,523 bp) in which the *matK* gene is present (Figure 1).

### Genome comparisons within Araliaceae

**Figure 2:**
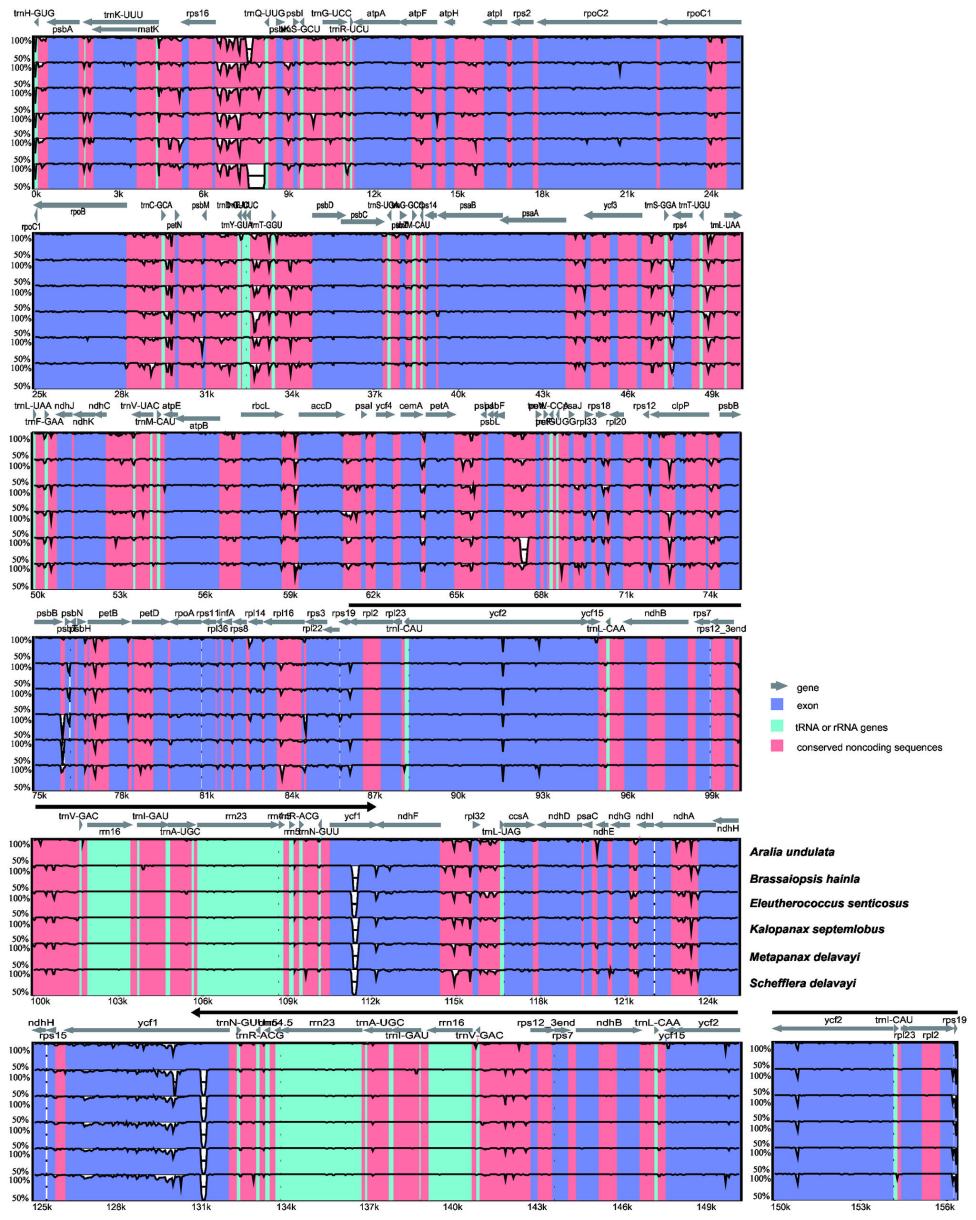
A percent identity plot showing the overall sequence similarity of the seven Araliaceae chloroplast genomes. Pair-wise comparison of chloroplast genomes within the Araliaceae using the mVISTA program with *Panax ginseng* as reference. The *Y* scale represents % identity ranging from 50–100%. Thick black lines show the inverted repeats (IRs) regions in the chloroplast genome. Genome regions are color-coded as protein coding, tRNA or rRNA genes, and conserved noncoding sequences.

Multiple complete chloroplast genomes of Araliaceae provide an opportunity to compare the sequence variation within the family. The sequence identity of all seven Araliaceae chloroplast genome was plotted using the mVISTA program with the annotation of *Panax ginseng* as the reference (Figure 2). The whole aligned sequences show high similarities with only a few regions with identities falling below 90%, suggesting that Araliaceae chloroplast genomes are rather conservative. Consistent with other angiosperms, the inverted repeat regions and coding regions are more conserved than the single copy and noncoding regions, respectively. The most conserved coding regions in Araliaceae genomes are the four ribosomal RNA genes. The *ycf1* gene is the most divergent coding region with lower sequence identity due to its various indels and highly variable sequences, which has been reported in other genomes [40]. The average genetic divergence of the seven Araliaceae species, estimated by *p*-distance, was only 0.008. The *p*-distance between the Asian Palmate group and the *Aralia*-*Panax* group was 0.011, which is higher than that within the Asian Palmate group (*p*-distance=0.005), but is nearly equal to that within the *Aralia*-*Panax* group (*p*-distance=0.01). These values show that sequence divergence in the *Aralia*-*Panax* group is much higher than that in the Asian Palmate group at the whole chloroplast genome level, even though the Asian Palmate group shows much higher taxonomic diversity consisting of about 20 genera, whereas the *Aralia*-*Panax* group has only two genera [1]. The diversification patterns of the two major clades will be explored in our future analyses.

### Contraction and expansion of inverted repeats (IRs)

**Figure 3:**
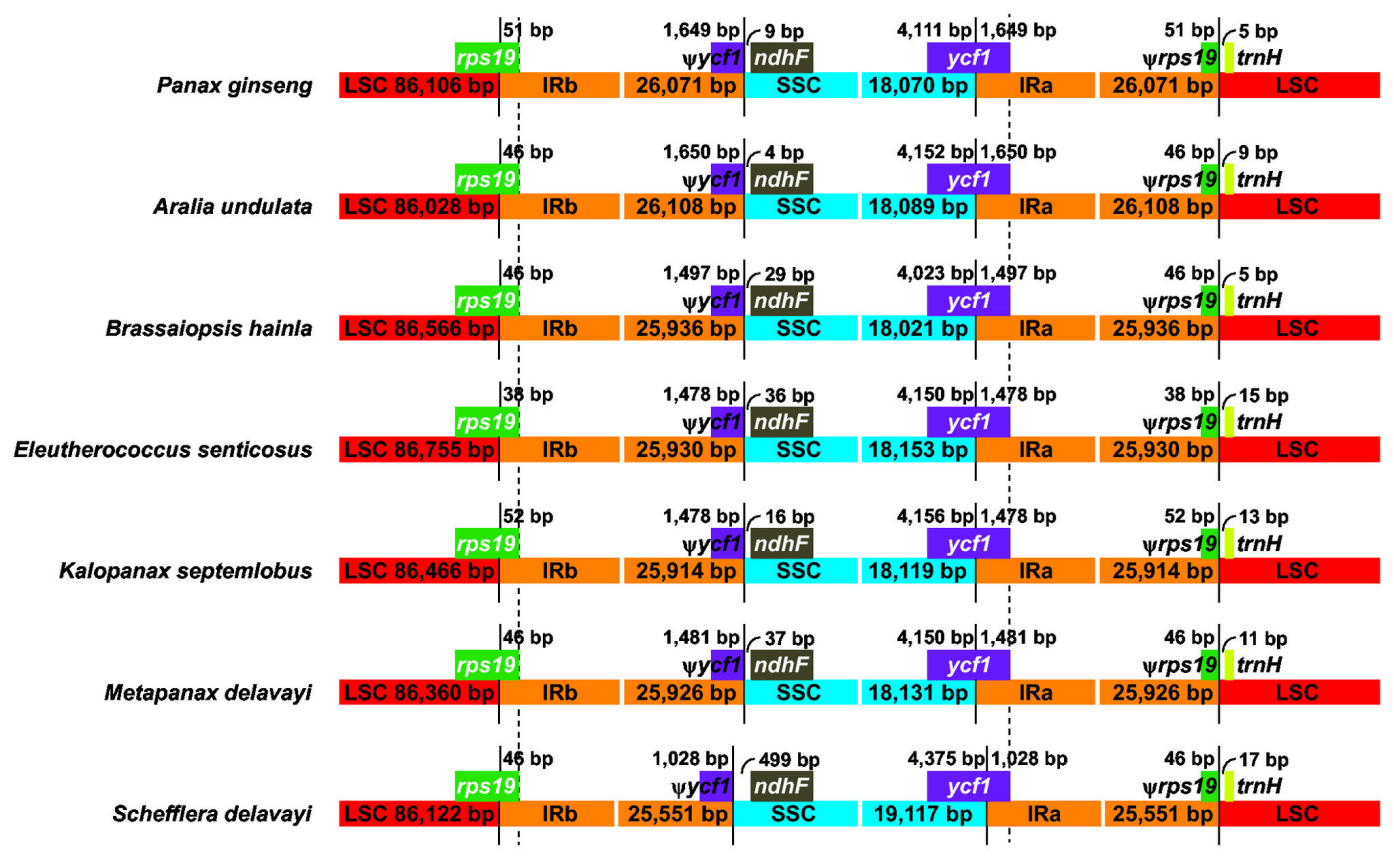
Comparison of border positions of LSC, SSC, and IR regions among seven Araliaceae chloroplast genomes. Boxes above the main line indicate the genes while the pseudogenes at the borders are shown by Ψ. The figure is not the scale and just shows relative changes at or near the IR/SC borders.

The expansion and contraction of the border region between the inverted repeats and the single-copy regions contribute to the major variation in length of the chloroplast genomes among plant lineages [28,50]. We compared exact IR/SC border positions and their adjacent genes among the seven Araliaceae chloroplast genomes (Figure 3). The IRb/LSC boundary structure in all these species was similar and located within the coding region of *rps19*, which resulted in the presence of the *rps19* pseudogenes in IRa with the same length as far as the IRb extended into the 5’portion of the *rps19* gene (38–52 bp). In addition, IR was further extended deep into the 3’ end of *ycf1* gene in all seven Araliaceae species and created the *ycf1* pseudogenes at IRb/SSC border with lengths of 1,028–1,650 bp. However, the *ycf1* gene extended less into the IR region in *Schefflera delavayi*, which partly explain why the SSC region in *Schefflera delavayi* is larger than that in other species (Figure 3).

The *ndhF* gene was entirely located in the SSC region in all the seven species but varied in distance from the IRb/SSC border. *Schefflera delavayi* has 499 bp, representing the longest space among these species, whereas *Aralia undulata* has only 4 bp (Figure 3). Significant structure divergence about the position of the *trnH* gene between monocots and dicots has been reported [51]. In general, the *trnH* gene is located in the IR region in the monocots, compared with its location in the LSC region in the dicots. The *trnH* gene of all seven Araliaceae chloroplast genomes is located in the LSC region and it is 5–17 bp apart from the IRa/LSC border (Figure 3).

### Repeat analysis

**Figure 4:**
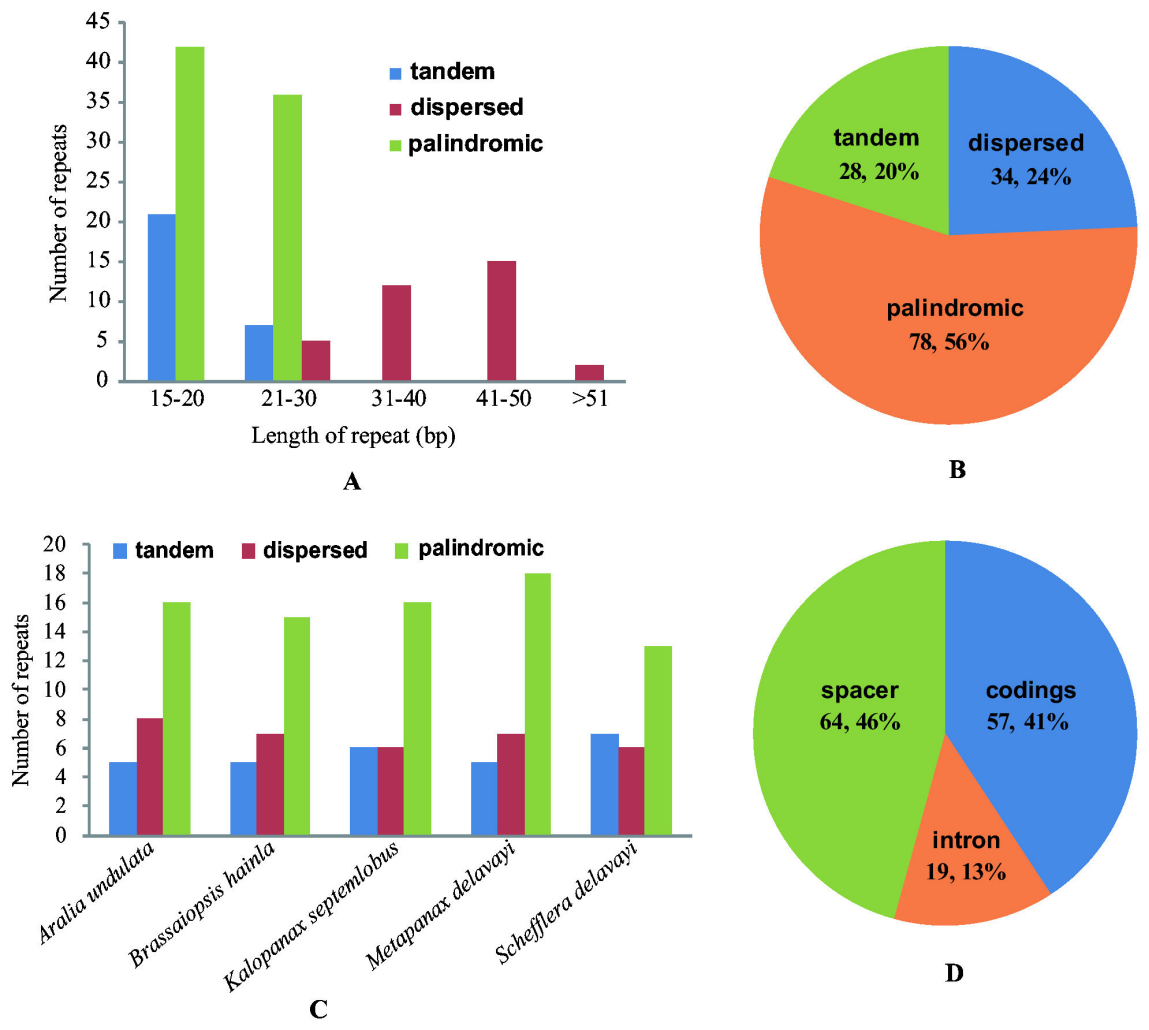
Repeat structure analysis in the five Araliaceae chloroplast genomes. A: Histogram showing the frequency of repeats by length in the five Araliaceae chloroplast genomes; B: Composition of the 140 repeats from five Araliaceae species; C: Histogram showing the number of three repeat types in each Araliaceae chloroplast genome; D: Location of the 140 repeats from five Araliaceae species.

In the current study, we divided the repeats into three categories: tandem, dispersed, and palindromic. A total of 140 repeats were identified in five Araliaceae chloroplast genomes (Table S2) using the program REPuter [39]. Seventy-eight palindromic repeats, accounting for 56% of total repeats, are the most common kind in the three types (Figure 4B). Most of these repeats (83 repeats, 59%) are located in noncoding regions (Figure 4D), while some are found in genes such as *psaB*, *ycf2*, *rpoC2*, *psbT*, and *atpA*. 79.3% of repeats range 15–30 bp in size (Figure 4A), although the defined smallest size is 20 bp and 30 bp for palindromic and dispersed repeats, respectively. The longest repeat is two dispersed repeats of 63 bp in *Aralia undulata*. Except for four 30 bp tandem repeats in *Aralia undulata* and *Schefflera delavayi*, all other tandem repeats are 25 bp or shorter, while palindromic repeats occur in a narrower size range from 20 to 30 bp. Numbers of the three repeat types are similar among the five chloroplast genomes (Figure 4C) and their overall distribution in the chloroplast genome is highly conserved (Table S2).

Repeat structure is considered to play an important role in the rearrangement of chloroplast genomes and generating divergent regions via illegitimate recombination and slipped-strand mispairing [28,51–55]. In our sequenced genomes, divergent regions are often associated with many repeat events. For example, the *ycf2* gene contains various repeats (Table S2).

### Phylogenetic implications

**Figure 5:**
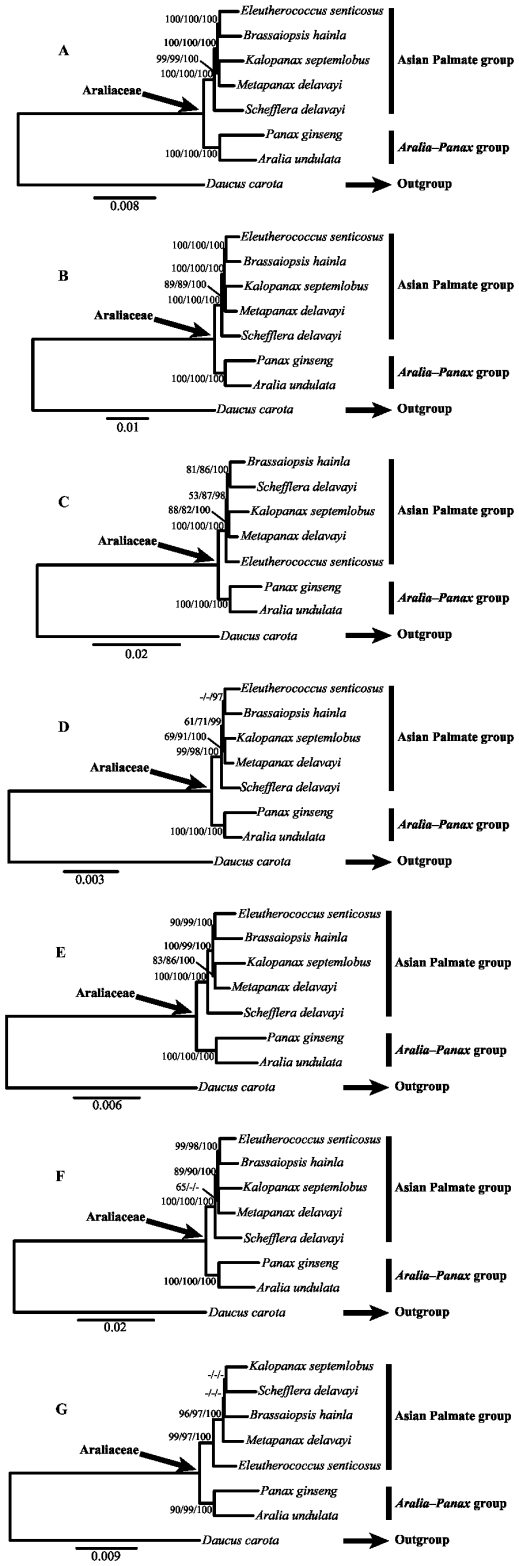
Phylogenetic relationships of Araliaceae based on seven different data partitions in chloroplast genomes. A: Complete chloroplast DNA sequences; B: The large single-copy (LSC) region; C: The small single-copy (SSC) region; D: The inverted repeat (IR) region; E: Eighty common protein coding genes; F: The intergenic spacers region; G: The introns region. Support values are shown for nodes as maximum parsimony bootstrap (PB)/maximum likelihood bootstrap (LB)/Bayesian inference posterior probability (PP). Dash shows that the PB value lower than 50% or the LB value lower than 70% or the PP value lower than 0.95. Branch lengths were calculated through Bayesian analysis, and scale bar denotes substitutions per site.

Seven data partitions (complete chloroplast DNA sequences, the LSC region, the SSC region, the IR region, protein coding genes, the intergenic spacers region, and the introns) from seven Araliaceae and one Apiaceae chloroplast genomes were used to construct phylogenetic trees (Table 4). Phylogenetic relationships with bootstrap values and posterior probabilities based on the seven data partitions are presented in Figure 5. The maximum parsimony analyses, maximum likelihood analyses, and Bayesian inference yielded the same topology in each data partition. Except the phylogenetic relationships were not resolved in the intron region tree, phylogenetic trees of the six other data partitions were largely congruent with each other (Figure 5). The only incongruence among the six trees is the positions of *Eleutherococcus senticosus* and *Schefflera delavayi*. In the SSC region tree, *Schefflera delavayi* is supported to be sister of *Brassaiopsis hainla* with moderate support, whereas *Eleutherococcus senticosus* as sister to *Brassaiopsis hainla* with strong support in five other trees (Figure 5). The shorter sequence length with fewer parsimony informative sites (PIS) in introns and SSC data partitions may account for the unresolved and incongruence. The best resolution in phylogenetic relationships was achieved using complete chloroplast DNA sequences, thus we discuss the phylogenetic relationships based on Figure 5A.

**Table 4:** Statistics of seven data partitions used in phylogenomic analyses.

**Analysis**	**Characters**	**Whole chloroplast genomes**	**LSC region**	**SSC region**	**IR region**	**Protein coding genes**	**Intergenic spacers**	**Introns**
Maximum parsimony	Aligned length (bp)	164,615	90,637	19,009	27,467	69,683	44,631	15,851
	Variable sites (%)	12,360 (7.51%)	8,294 (9.15%)	2,533 (13.33%)	769 (2.80%)	4,376 (6.28%)	5,767 (12.92%)	1,373 (8.66%)
	Informative sites (%)	1,164 (0.71%)	776 (0.86%)	309 (1.63%)	39 (0.14%)	464 (0.67%)	497 (1.11%)	156 (0.98%)
	Tree length	13,503	9,027	2,889	791	4,801	6,292	1,528
	Consistency index (CI)	0.9511	0.9523	0.9339	0.9823	0.9488	0.9541	0.9359
	Retention index (RI)	0.5728	0.5864	0.5201	0.7308	0.6019	0.5533	0.5442
Maximum likelihood	-lnL	299568.231123	171416.691615	39791.133899	42835.819761	122770.698809	90158.672476	29847.663050
Bayesian inference	Model selected by AIC	TVM+I+G	GTR+I+G	TVM+I+G	TIM+I	TVM+I+G	TVM+G	TIM+I+G

In our study, the Asian Palmate group and the *Aralia*-*Panax* group were strongly supported as a monophyletic group, respectively (PB=100%, LB=100%, PP=100%). It is the first successful attempt to provide phylogenomic analyses for the relationships within Araliaceae based on chloroplast genomes, and the results were consistent with those of previous phylogenetic studies with selected DNA fragments and broad sampling [1–3].

Within the Asian Palmate group (Figure 5A), *Schefflera delavayi* is the earliest diverging lineage, which was identified as sister to the other species in this group with strong support (PB=100%, LB=100%, PP=100%). Also, *Eleutherococcus senticosus* + *Brassaiopsis hainla* and *Kalopanax septemlobus* + *Metapanax delavayi* formed a clade, respectively, with the two clades sister to each other (PB=100%, LB=100%, PP=100%). This result suggests that the lack of sufficient characters are the main reason to the poor phylogenetic resolution within the Asian Palmate group in previous studies [1–3]. However, we only included five genera of the Asian Palmate group in this study and insufficient taxon sampling has been known to result in misleading conclusions [56–59]. Therefore, more complete chloroplast genome sequences of the Asian Palmate group are necessary to confirm the phylogenetic relationships within the group. On the other hand, the improved phylogenetic resolution indicated that phylogenomics based on complete chloroplast genomes can be useful for resolving the relationships of complex lineages with a rapid diversification history.

Most phylogenomic studies used common protein coding genes [22,23,60]. In this study, the support values for each node in phylogenetic trees were reduced by using data partitions of the SSC region (Figure 5C), the IR region (Figure 5D), the protein coding genes (Figure 5E), the intergenic spacers region (Figure 5F), and the introns (Figure 5G), which contained fewer PIS than the other two data partitions (Table 4). This result indicated that at least the LSC region was needed to provide good resolution for the sampled taxa. In our study, complete chloroplast DNA sequences were proved to be more effective than common protein coding genes for the phylogenetic reconstruction of Araliaceae, as evaluated by bootstrap values and posterior probabilities. Therefore, we suggest that complete chloroplast genomes, or even just the LSC region, could be used for constructing the backbones relationships among main clades, as well as for solving the phylogenetic positions of some critical lineages.

**Figure 6:**
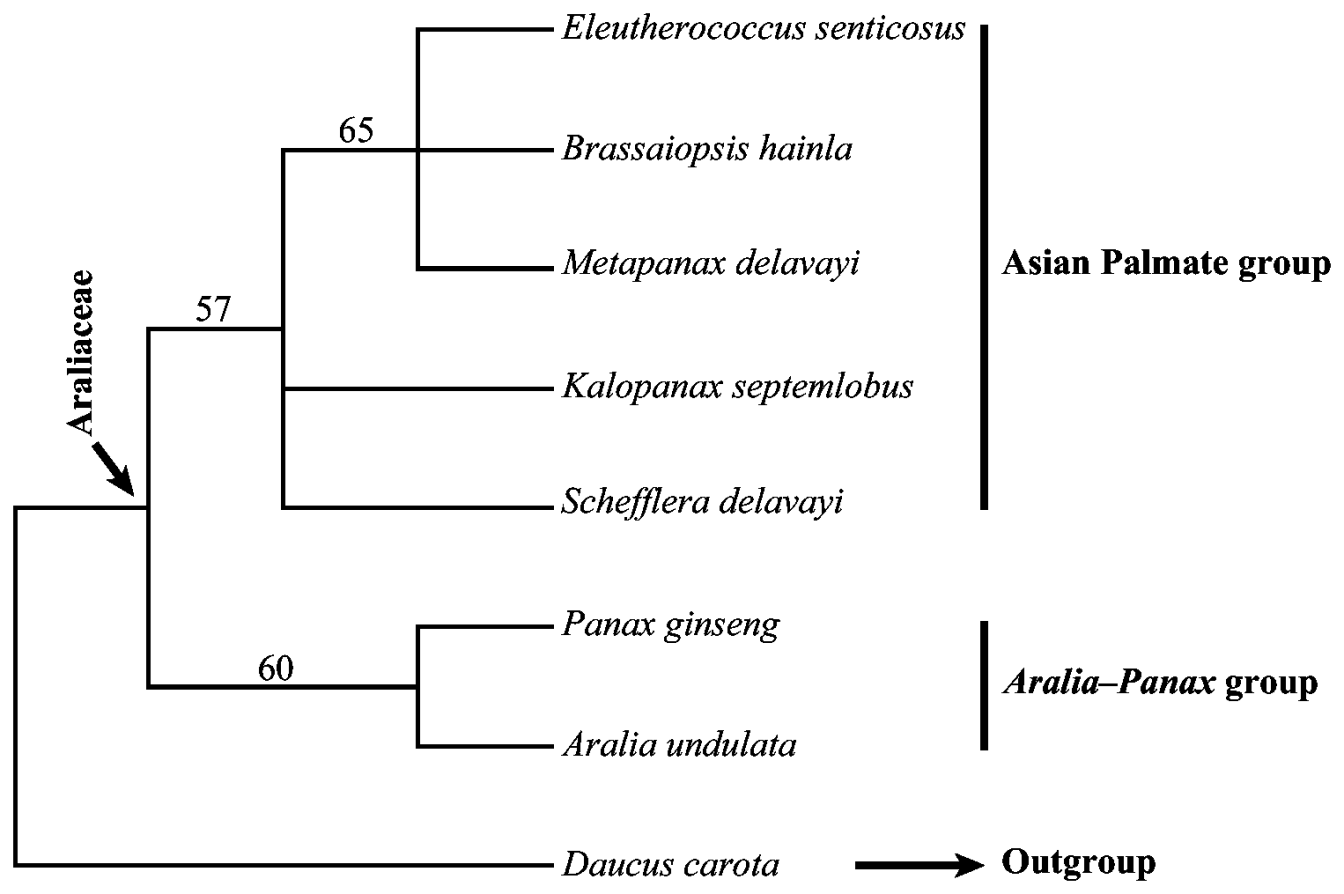
Phylogeny reconstructed using the indels from exon of coding regions in seven Araliaceae species. The numbers above branches indicate parsimony bootstrap values (PB) for maximum parsimony analysis.

Whether indels should be used for phylogenetic analyses has been debated [61,62]. In our study, 65 potentially informative indels from exons in 18 genes were identified (Table S3) and used to construct phylogenetic tree (Figure 6). The resulting topology was similar to the phylogenetic tree of complete chloroplast DNA sequences. Because of the limited informative sites, the Asian Palmate group and the *Aralia*-*Panax* group were only weakly supported as a monophyletic group, and the relationships within the Asian Palmate group were not well resolved. Thus, we suggest that minor chloroplast genome structural changes such as indels should be used cautiously in phylogenetic studies.

### Genome evolutionary patterns and DNA marker identification

**Figure 7:**
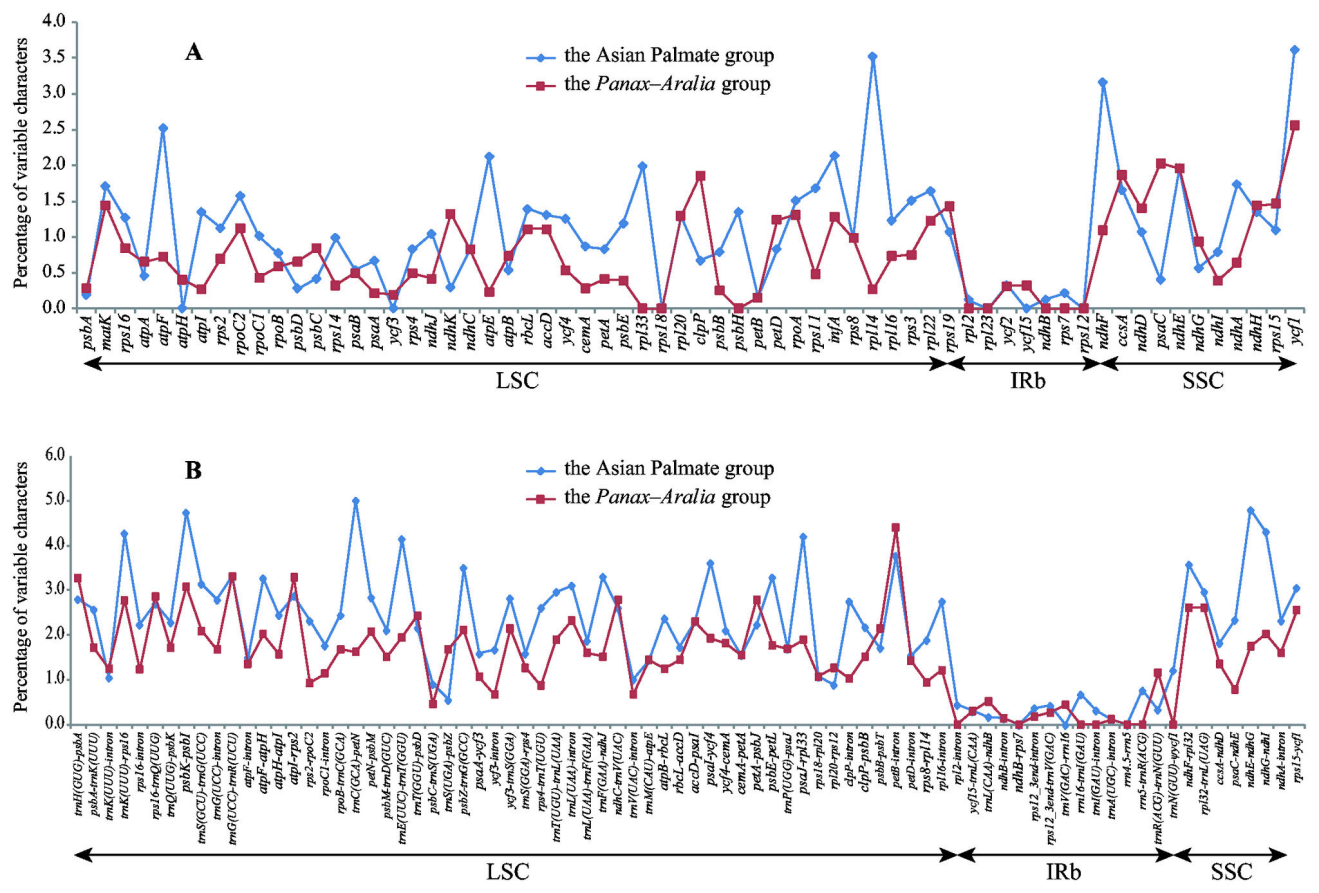
Percentage of variable characters in homologous regions between the chloroplast genomes of the Asian Palmate group and the *Panax-Aralia* group. A: Coding region; B: Noncoding region. The homologous regions are oriented according to their locations in the chloroplast genome.

It is critical to examine the genomic evolutionary patterns between the Asian Palmate group and the *Aralia*-*Panax* group by the comparative genomics, because low genetic divergence between the two groups was detected in our study. Based on the coding and noncoding regions longer than 200 bp, we found that the Asian Palmate group accumulated more mutations in their chloroplast genomes than the *Aralia*-*Panax* group as indicated by percentage of variations (Figure 7). The number and distribution pattern of variable characters in coding and noncoding regions were rather different between the Asian Palmate group and the *Aralia*-*Panax* group. For example, *trnC*(*GCA*)*-petN* accumulated more variations than other noncoding regions in the Asian Palmate group. However, it was not the most variable region (in terms of the percentage of variation) in the *Aralia*-*Panax* group. Thus, the nucleotide substitution pattern of each region is different in the two groups.

Phylogenetic relationships within Araliaceae have not been well resolved in previous studies, which may be due to the low rate of nucleotide substitutions in the selected DNA markers for phylogenetic analyses [1,3]. It is necessary to employ more rapidly evolving regions for phylogenetic studies of Araliaceae. Correlation analysis revealed a significant positive linear relationship (R^2^ = 0.3914, P < 0.0001) between percentage of parsimony informative sites and percentage of variable sites in candidate regions. Therefore, we have selected 26 most variable regions as potential phylogenetic markers (Table S4). The percentage of variable sites for the 26 regions exceeds 5%, with the mean percentage of parsimony informative sites as 1.12%. Except that the *ycf1* gene is from coding region, all the other 25 markers identified are from noncoding regions. The 26 identified regions in this study are listed as following with from high to low genetic divergence: *trnK*(*UUU*)*-rps16*, *trnC*(*GCA*)*-petN*, *psbK-psbI*, *trnE*(*UUC*)*-trnT*(*GGU*), *ycf1*, *rps16-trnQ*(*UUG*), *psaJ-rpl33*, *ndhF-rpl32*, *trnT*(*UGU*)*-trnL*(*UAA*), *rps15-ycf1*, *rpl32-trnL*(*UAG*), *petB-intron*, *trnS*(*GCU*)*-trnG*(*UCC*), *ycf3-trnS*(*GGA*), *ndhG-ndhI*, *psaI-ycf4*, *trnH*(*GUG*)*-psbA*, *ndhC-trnV*(*UAC*), *trnL*(*UAA*)*-intron*, *trnF*(*GAA*)*-ndhJ*, *accD-psaI*, *atpF-atpH*, *petN-psbM*, *psbE-petL*, *petA-psbJ*, *ndhA-intron*. Six of them are located in SSC region (*ycf1*, *ndhF-rpl32*, *rps15-ycf1*, *rpl32-trnL*(*UAG*), *ndhG-ndhI*, *ndhA-intron*). Our current study newly identified 16 DNA regions (Table S4), in comparison with a previous study on DNA barcodes [63]. Interestingly, 22 intergenic spacers of these identified regions have been used in evolutionary studies of major *Panax* species [64].

To examine phylogenetic applications for the twenty-six fast evolving DNA regions, a maximum parsimony tree was constructed for each molecular marker in seven Araliaceae species (Figure S1). The results revealed that none of these regions alone was efficient to resolve the relationships among the current samples. Further studies with a broad sampling scheme need to be conducted to test the efficiency of these 26 identified regions in phylogenetic analysis of Araliaceae.

## Conclusion

We obtained the complete sequences of five Araliaceae chloroplast genomes using the Illumina sequencing-by-synthesis technology. All Araliaceae chloroplast genomes are similar in gene size, gene content, gene order, AT content, and IR/SC boundary structure. Three repeat types were investigated in each chloroplast genome, whose number and distribution are similar among the five chloroplast genomes. Phylogenomic analyses based on eight complete chloroplast genomes from the Araliaceae and Apiaceae provided strong support for the monophyly of the Asian Palmate group and the *Aralia*-*Panax* group. Furthermore, the relationships among the sampled taxa within the Asian Palmate group were well resolved. The complete chloroplast DNA sequences were shown to be effective for the phylogenetic reconstruction of Araliaceae. Differences in genome evolutionary pattern for each region have been observed between the Asian Palmate group and the *Aralia*-*Panax* group. Twenty-six fast evolving DNA regions were identified for future phylogenetic studies of Araliaceae.

## Supporting Information

Figure S1 **Maximum parsimony trees of 26 fast evolving DNA regions in seven Araliaceae species.** The numbers above branches indicate parsimony bootstrap values (PB) for maximum parsimony analysis.
(PDF)

Table S1 **Primers used for gap closure and junction verification.**
(DOCX)

Table S2 **Repeat sequences in the five Araliaceae chloroplast genomes.**
(DOCX)

Table S3 **Indels in exons of genes in the seven Araliaceae chloroplast genomes.**
(DOCX)

Table S4 **Summary statistics for candidate regions in the chloroplast genomes of Araliaceae.**
(DOCX)
