# Supplementary figures and images for: Complete Sequencing of Five Araliaceae Chloroplast Genomes and the Phylogenetic Implications
Source: PLoS One. 2013 Oct 18;8(10):e78568. doi: 10.1371/journal.pone.0078568 (PMC3799623; doi:10.1371/journal.pone.0078568)

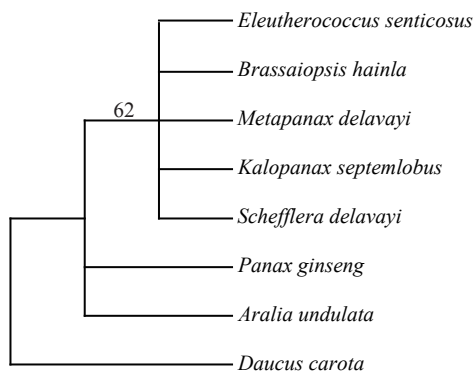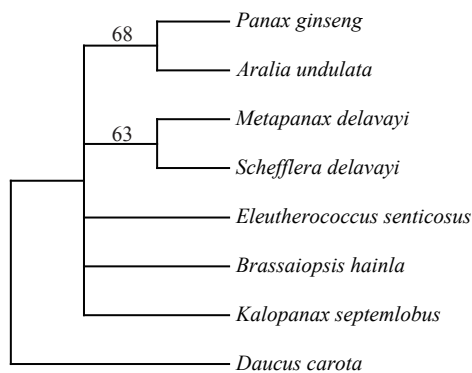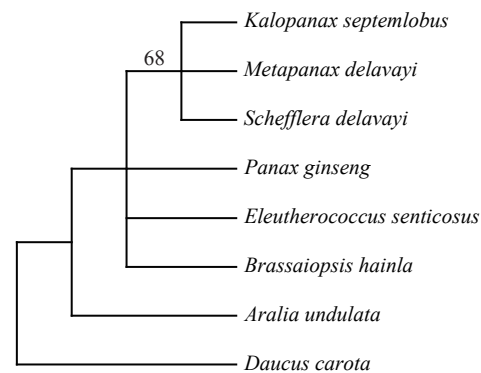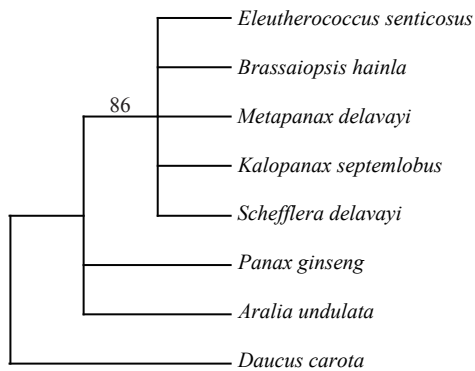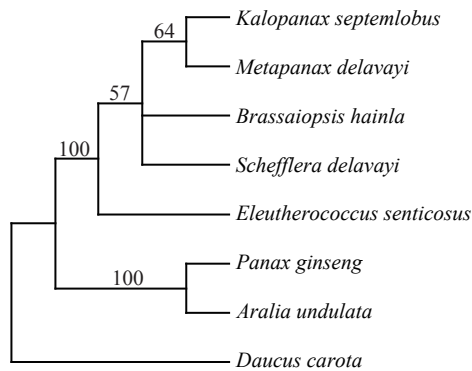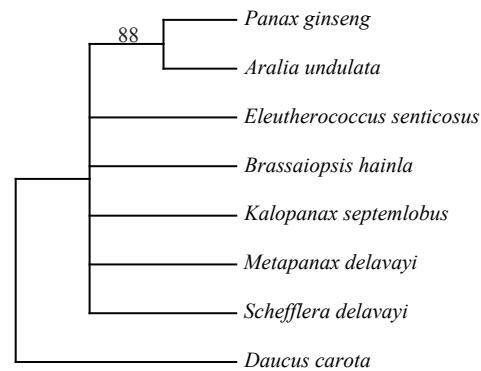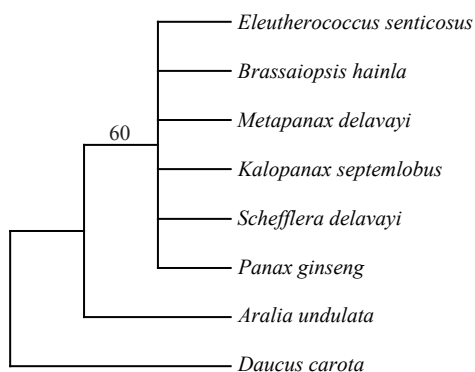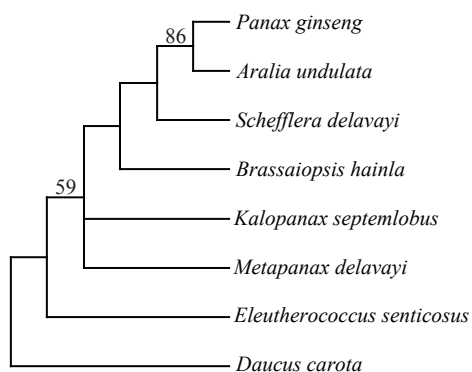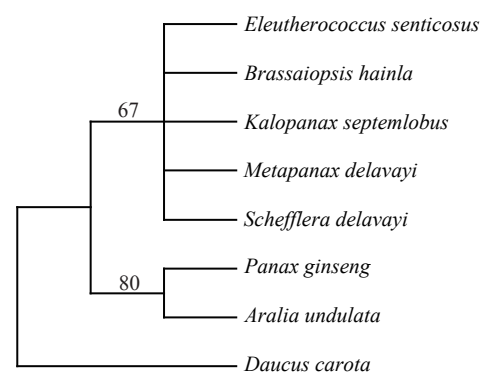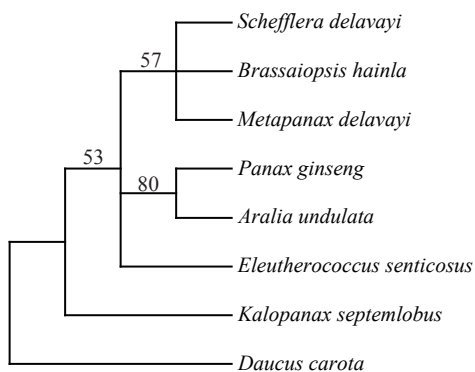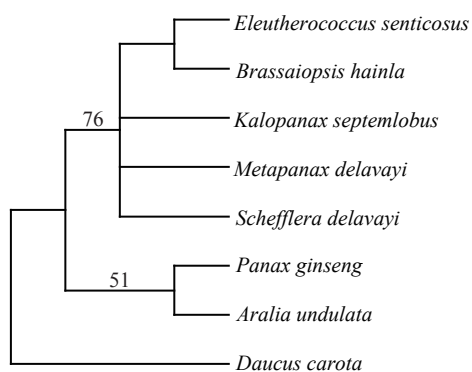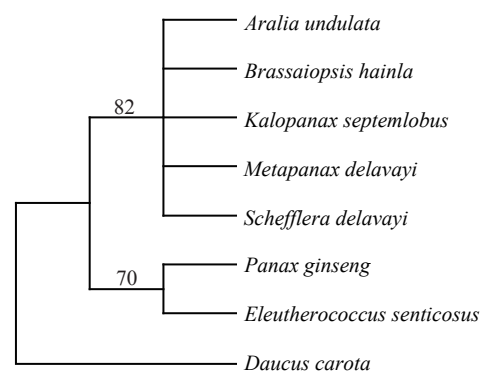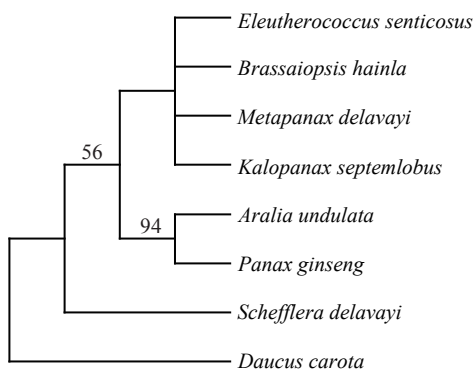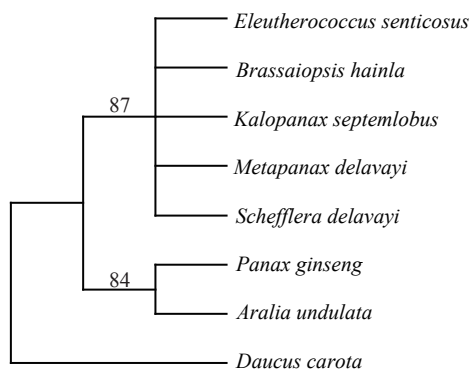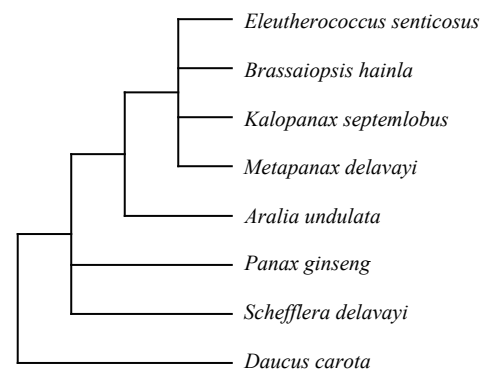

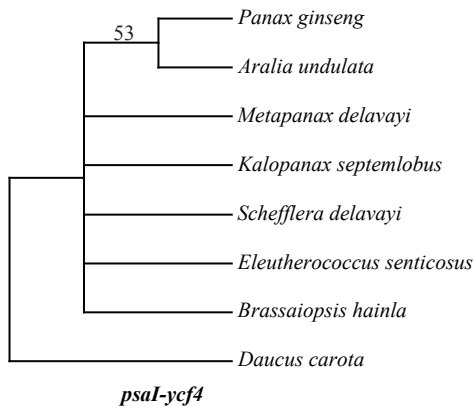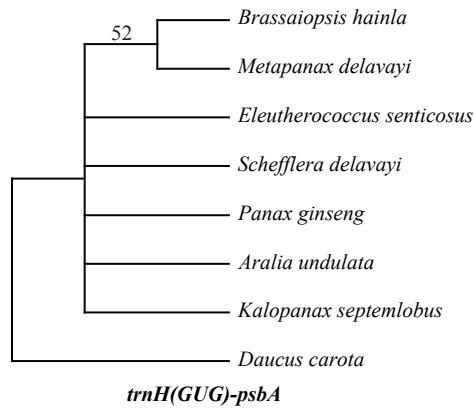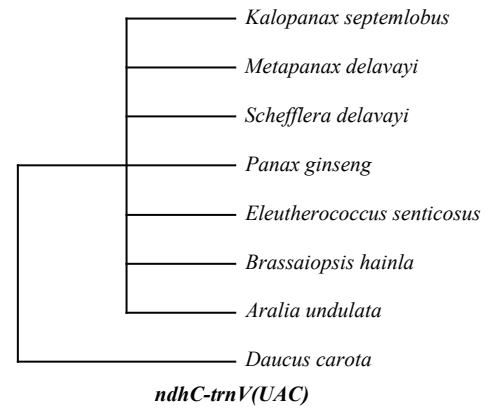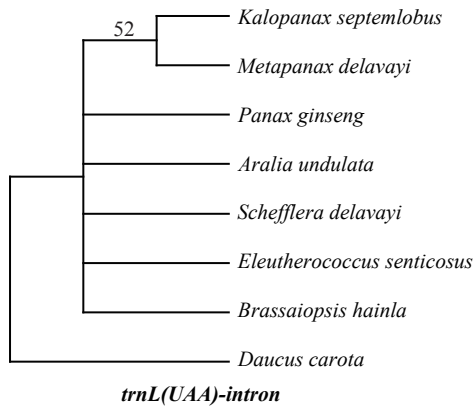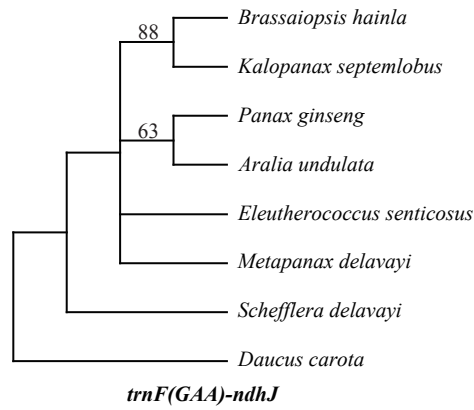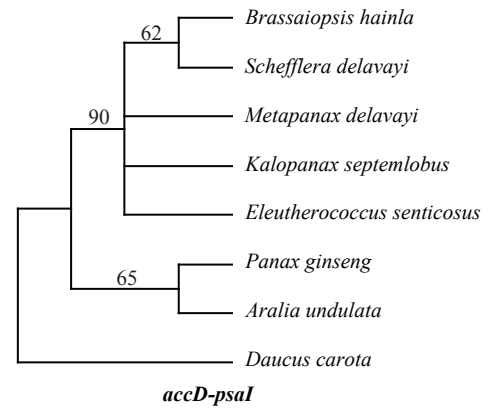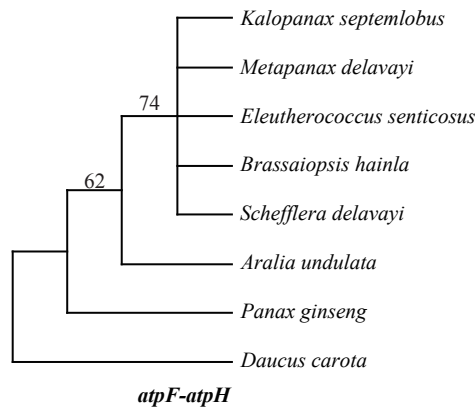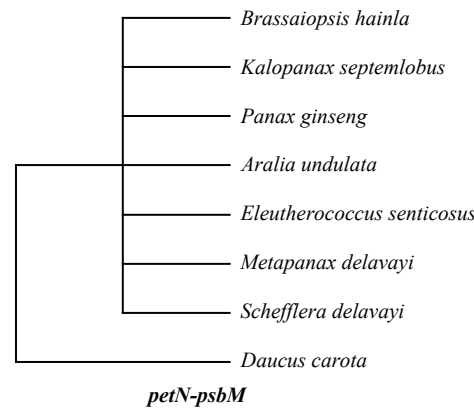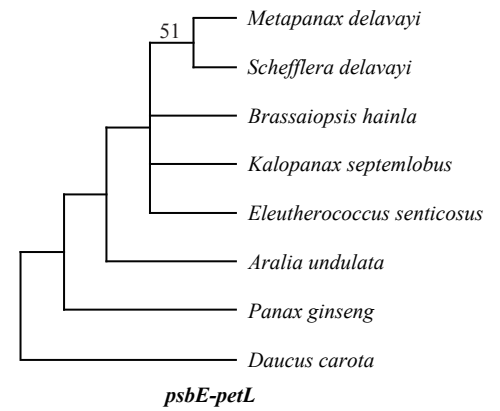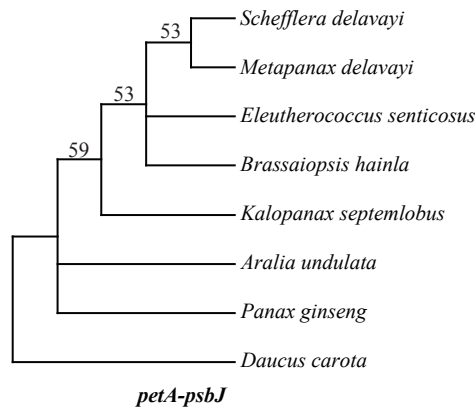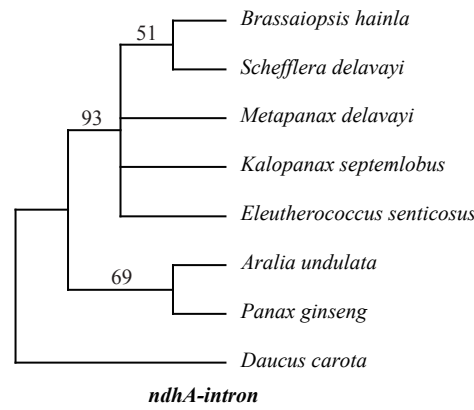

Supplement: Figure S1 — Maximum parsimony trees of 26 fast evolving DNA regions in seven Araliaceae species. The numbers above branches indicate parsimony bootstrap values (PB) for maximum parsimony analysis. (PDF) [file pone.0078568.s001.pdf]
