# Supplementary material for: Complete Sequencing of Five Araliaceae Chloroplast Genomes and the Phylogenetic Implications
Source: PLoS One. 2013 Oct 18;8(10):e78568. doi: 10.1371/journal.pone.0078568 (PMC3799623; doi:10.1371/journal.pone.0078568)
Supplement: Table S1 — Primers used for gap closure and junction verification. (DOCX) [file pone.0078568.s002.docx]

**Table S1.** Primers used for gap closure and junction verification.

| **Primer^*^** | **Sequence (5’>3’)** | **Gaps size or amplified size of junctions^**^** |
| --- | --- | --- |
| 1 | F: TTCATTCCCTGYAGGCCCGT  R: CTGACATGTCGCTTGGRAGG | 56 |
| 2 | F: TCCATTAGAAGGGGCTCGCA  R: CTTATGGCAGAACGGGCCAA | 332 |
| 3 | F: CCCCCGCTAACCCGAGTGAA  R: CCCTGGAGGATTGACAGGGCGA | 627 |
| 4 | F: CGGGTGTCGCCTGATCAACA  R: TGCGCAAWATGTGACTCGCCT | 334 |
| 5 | F: CCTGGATAAGCTTCGCGACC  R: KCATCTTGGGGGCGATGAAA | 489 |
| 6 | F: CAGTCCGTCCCCATTAACCG  R: ACGCCTTACCATGGCGTTAC | 623 |
| 7 | F: GGAGACCCACGTTCTACCGA  R: TAATACCGGTGCCACGGAGA | 246 |
| 8 | F: CCCTYGCTGACTTCAGCTTT  R: AGTGGGGAATGTTGGGGTGA | 4430 |
| 9 | F: AGTGGGGAATGTTGGGGTGA  R: AGAGGGCGGTATTGCTCCTT | 3676 |
| 10 | F: ACGATGGAATCGCCCATTACG  R: TGTGGTATTCCGCCTCTTGC | 6737 |
| 11 | F: GCCCGWCTGTTGTTCCAACT  R: AGGCAGAATACCGTCACCCA | 6237 |

^*^ Primer pairs 1, 2-3, and 4-7 were used to finish gaps in the assembly of *Aralia undulata*, *Brassaiopsis hainla*, and *Schefflera delavayi*, respectively; Primer pairs 8-11 were used to verify the J­­_LB_, J_LA_, J_SB_, and J_SA_, respectively.

^**^ Amplified size of junctions was the total size amplified in the five chloroplast genomes.
