# Supplementary material for: Complete Sequencing of Five Araliaceae Chloroplast Genomes and the Phylogenetic Implications
Source: PLoS One. 2013 Oct 18;8(10):e78568. doi: 10.1371/journal.pone.0078568 (PMC3799623; doi:10.1371/journal.pone.0078568)
Supplement: Table S2 — Repeat sequences in the five Araliaceae chloroplast genomes. (DOCX) [file pone.0078568.s003.docx]

**Table S2.** Repeat sequences in the five Araliaceae chloroplast genomes.

| **Genomes^*^** | **Repeat types** | **Length (bp)** | **Position A** | **Position B** | **Location** |
| --- | --- | --- | --- | --- | --- |
| AU | tandem | 25 | 59129 | 59154 | *rbcL-accD* |
| AU | tandem | 19 | 76097 | 76116 | *psbT* |
| AU | tandem | 30 | 91532 | 91562 | *ycf2* |
| AU | tandem | 18 | 122688 | 122706 | *ndhA-intron* |
| AU | tandem | 30 | 150770 | 150800 | *ycf2* |
| AU | dispersed | 31 | 9167 | 37505 | *psbI-trnS(GCU); psbC-trnS(UGA)* |
| AU | dispersed | 30 | 40669 | 42893 | *psaB; psaA* |
| AU | dispersed | 43 | 92729 | 92783 | *ycf2* |
| AU | dispersed | 42 | 99817 | 122533 | *rps12(3end)-trnV(GAC); ndhA-intron* |
| AU | dispersed | 31 | 109194 | 133135 | *rrn5-trnR(ACG); trnR(ACG)-rrn5* |
| AU | dispersed | 63 | 111234 | 111405 | *ycf1* |
| AU | dispersed | 63 | 130892 | 131063 | *ycf1* |
| AU | dispersed | 43 | 149536 | 149590 | *ycf2* |
| AU | palindromic | 20 | 1722 | 3789 | *psbA-trnK(UUU); trnK(UUU)-intron* |
| AU | palindromic | 20 | 4103 | 4769 | *trnK(UUU)-intron; trnK(UUU)-rps16* |
| AU | palindromic | 20 | 7107 | 7156 | *rps16-trnQ(UUG)* |
| AU | palindromic | 20 | 28284 | 29091 | *rpoB-trnC(GCA)* |
| AU | palindromic | 23 | 30786 | 30819 | *petN-psbM* |
| AU | palindromic | 21 | 32779 | 32803 | *trnE(UUC)-trnT(GGU)* |
| AU | palindromic | 21 | 48383 | 48814 | *rps4-trnT(UGU); trnT(UGU)-trnL(UAA)* |
| AU | palindromic | 20 | 72547 | 74259 | *clpP-intron; clpP-psbB* |
| AU | palindromic | 21 | 76992 | 77056 | *petB-intron* |
| AU | palindromic | 24 | 78611 | 78637 | *petD-intron* |
| AU | palindromic | 20 | 89874 | 92159 | *ycf2* |
| AU | palindromic | 20 | 92102 | 92234 | *ycf2* |
| AU | palindromic | 22 | 95212 | 95238 | *ycf15-trnL(CAA)* |
| AU | palindromic | 22 | 147102 | 147128 | *trnL(CAA)-ycf15* |
| AU | palindromic | 20 | 150108 | 150240 | *ycf2* |
| AU | palindromic | 20 | 150183 | 152468 | *ycf2* |
| BH | tandem | 25 | 59560 | 59585 | *rbcL-accD* |
| BH | tandem | 16 | 65621 | 65637 | *petA-psbJ* |
| BH | tandem | 17 | 69377 | 69394 | *trnP(UGG)-psaJ* |
| BH | tandem | 15 | 92086 | 92101 | *ycf2* |
| BH | tandem | 15 | 150910 | 150925 | *ycf2* |
| BH | dispersed | 31 | 9351 | 37744 | *psbI-trnS(GCU); psbC-trnS(UGA)* |
| BH | dispersed | 32 | 20653 | 20686 | *rpoC2* |
| BH | dispersed | 30 | 40907 | 43131 | *psaB; psaA* |
| BH | dispersed | 43 | 93253 | 93307 | *ycf2* |
| BH | dispersed | 42 | 100335 | 122972 | *rps12(3end)-trnV(GAC); ndhA-intron* |
| BH | dispersed | 31 | 109715 | 133280 | *rrn5-trnR(ACG); trnR(ACG)-rrn5* |
| BH | dispersed | 43 | 149676 | 149730 | *ycf2* |
| BH | palindromic | 20 | 4081 | 4760 | *trnK(UUU)-intron; trnK(UUU)-rps16* |
| BH | palindromic | 20 | 11211 | 11332 | *trnR(UCU)-atpA; atpA* |
| BH | palindromic | 21 | 29838 | 29864 | *trnC(GCA)-petN* |
| BH | palindromic | 27 | 30992 | 31029 | *petN-psbM* |
| BH | palindromic | 20 | 44681 | 47479 | *psaA-ycf3; ycf3-trnS(GGA)* |
| BH | palindromic | 30 | 61868 | 64105 | *accD-psaI; cemA-petA* |
| BH | palindromic | 21 | 77516 | 77576 | *petB-intron* |
| BH | palindromic | 24 | 79130 | 79158 | *petD-intron* |
| BH | palindromic | 20 | 90428 | 92683 | *ycf2* |
| BH | palindromic | 20 | 92626 | 92758 | *ycf2* |
| BH | palindromic | 22 | 95728 | 95754 | *ycf15-trnL(CAA)* |
| BH | palindromic | 20 | 128218 | 128533 | *ycf1* |
| BH | palindromic | 22 | 147250 | 147276 | *trnL(CAA)-ycf15* |
| BH | palindromic | 20 | 150248 | 150380 | *ycf2* |
| BH | palindromic | 20 | 150323 | 152578 | *ycf2* |
| KS | tandem | 20 | 14219 | 14239 | *atpF-atpH* |
| KS | tandem | 15 | 30936 | 30951 | *petN-psbM* |
| KS | tandem | 16 | 50929 | 50945 | *trnF(GAA)-ndhJ* |
| KS | tandem | 17 | 69382 | 69399 | *trnP(UGG)-psaJ* |
| KS | tandem | 15 | 91980 | 91995 | *ycf2* |
| KS | tandem | 15 | 150870 | 150885 | *ycf2* |
| KS | dispersed | 31 | 9406 | 37737 | *psbI-trnS(GCU); psbC-trnS(UGA)* |
| KS | dispersed | 30 | 40917 | 43141 | *psaB; psaA* |
| KS | dispersed | 43 | 93147 | 93201 | *ycf2* |
| KS | dispersed | 42 | 100229 | 122838 | *rps12(3end)-trnV(GAC); ndhA-intron* |
| KS | dispersed | 31 | 109612 | 133237 | *rrn5-trnR(ACG); trnR(ACG)-rrn5* |
| KS | dispersed | 43 | 149636 | 149690 | *ycf2* |
| KS | palindromic | 20 | 4131 | 4828 | *trnK(UUU)-intron; trnK(UUU)-rps16* |
| KS | palindromic | 20 | 11107 | 12894 | *trnG(UCC)-trnR(UCU); atpA-atpF* |
| KS | palindromic | 20 | 11278 | 11399 | *trnR(UCU)-atpA; atpA* |
| KS | palindromic | 27 | 31043 | 31080 | *psbM* |
| KS | palindromic | 20 | 44685 | 47479 | *psaA-ycf3; ycf3-trnS(GGA)* |
| KS | palindromic | 20 | 61917 | 64120 | *accD-psaI; cemA-petA* |
| KS | palindromic | 21 | 73064 | 74790 | *clpP-intron; clpP-psbB* |
| KS | palindromic | 21 | 77445 | 77505 | *petB-intron* |
| KS | palindromic | 24 | 79069 | 79095 | *petD-intron* |
| KS | palindromic | 20 | 90322 | 92577 | *ycf2* |
| KS | palindromic | 20 | 92520 | 92652 | *ycf2* |
| KS | palindromic | 22 | 95622 | 95648 | *ycf15-trnL(CAA)* |
| KS | palindromic | 20 | 128062 | 128377 | *ycf1* |
| KS | palindromic | 22 | 147210 | 147236 | *trnL(CAA)-ycf15* |
| KS | palindromic | 20 | 150208 | 150340 | *ycf2* |
| KS | palindromic | 20 | 150283 | 152538 | *ycf2* |
| MD | tandem | 17 | 69113 | 69130 | *trnP(UGG)-psaJ* |
| MD | tandem | 19 | 76395 | 76414 | *psbT* |
| MD | tandem | 15 | 91880 | 91895 | *ycf2* |
| MD | tandem | 17 | 112290 | 112307 | *ycf1* |
| MD | tandem | 15 | 150794 | 150809 | *ycf2* |
| MD | dispersed | 31 | 9340 | 37763 | *psbI-trnS(GCU); psbC-trnS(UGA)* |
| MD | dispersed | 30 | 40935 | 43159 | *psaB; psaA* |
| MD | dispersed | 35 | 45942 | 97068 | *ycf3-intron; ndhB-intron* |
| MD | dispersed | 43 | 93047 | 93101 | *ycf2* |
| MD | dispersed | 42 | 100129 | 122750 | *rps12(3end)-trnV(GAC); ndhA-intron* |
| MD | dispersed | 31 | 109518 | 133155 | *rrn5-trnR(ACG); trnR(ACG)-rrn5* |
| MD | dispersed | 43 | 149560 | 149614 | *ycf2* |
| MD | palindromic | 20 | 4089 | 4742 | *trnK(UUU)-intron; trnK(UUU)-rps16* |
| MD | palindromic | 21 | 6507 | 9058 | *rps16-trnQ(UUG); psbK-psbI* |
| MD | palindromic | 20 | 11209 | 11330 | *trnR(UCU)-atpA; atpA* |
| MD | palindromic | 27 | 30972 | 31009 | *petN-psbM* |
| MD | palindromic | 22 | 33035 | 33060 | *trnE(UUC)-trnT(GGU)* |
| MD | palindromic | 20 | 44703 | 47500 | *psaA-ycf3; ycf3-trnS(GGA)* |
| MD | palindromic | 20 | 61862 | 64066 | *accD-psaI; cemA-petA* |
| MD | palindromic | 20 | 67627 | 67671 | *psbE-petL* |
| MD | palindromic | 21 | 72813 | 74555 | *clpP-intron; clpP-psbB* |
| MD | palindromic | 24 | 78919 | 78945 | *petD-intron* |
| MD | palindromic | 20 | 90222 | 92477 | *ycf2* |
| MD | palindromic | 20 | 92420 | 92552 | *ycf2* |
| MD | palindromic | 22 | 95522 | 95548 | *ycf15-trnL(CAA)* |
| MD | palindromic | 20 | 119583 | 119604 | *ndhD-psaC* |
| MD | palindromic | 20 | 127986 | 128301 | *ycf1* |
| MD | palindromic | 22 | 147134 | 147160 | *trnL(CAA)-ycf15* |
| MD | palindromic | 20 | 150132 | 150264 | *ycf2* |
| MD | palindromic | 20 | 150207 | 152462 | *ycf2* |
| SD | tandem | 21 | 10453 | 10474 | *trnG(UCC)-trnR(UCU)* |
| SD | tandem | 18 | 31599 | 31617 | *psbM-trnD(GUC)* |
| SD | tandem | 17 | 68832 | 68849 | *trnP(UGG)-psaJ* |
| SD | tandem | 17 | 75872 | 75889 | *psbB-psbT* |
| SD | tandem | 19 | 76125 | 76144 | *psbT* |
| SD | tandem | 30 | 91653 | 91683 | *ycf2* |
| SD | tandem | 30 | 150751 | 150781 | *ycf2* |
| SD | dispersed | 31 | 8792 | 37201 | *psbI-trnS(GCU); psbC-tnrS(UGA)* |
| SD | dispersed | 30 | 40369 | 42593 | *psaB; psaA* |
| SD | dispersed | 43 | 92850 | 92904 | *ycf2* |
| SD | dispersed | 42 | 99950 | 122618 | *rps12(3end)-trnV(GAC); ndhA-intron* |
| SD | dispersed | 31 | 109343 | 133090 | *rrn5-trnR(ACG); trnR(ACG)-rrn5* |
| SD | dispersed | 43 | 149517 | 149571 | *ycf2* |
| SD | palindromic | 20 | 10689 | 10810 | *trnR(UCU)-atpA; atpA* |
| SD | palindromic | 26 | 29268 | 29297 | *trnC(GCA)-petN* |
| SD | palindromic | 24 | 30428 | 30468 | *petN-psbM* |
| SD | palindromic | 21 | 72529 | 74264 | *clpP-intron; clpP-psbB* |
| SD | palindromic | 21 | 77030 | 77090 | *petB-intron* |
| SD | palindromic | 24 | 78647 | 78673 | *petD-intron* |
| SD | palindromic | 24 | 83762 | 83826 | *rpl16-intron* |
| SD | palindromic | 20 | 92223 | 92355 | *ycf2* |
| SD | palindromic | 22 | 95343 | 95369 | *ycf15-trnL(CAA)* |
| SD | palindromic | 23 | 114892 | 114922 | *ndhF-rpl32* |
| SD | palindromic | 22 | 147073 | 147099 | *trnL(CAA)-ycf15* |
| SD | palindromic | 20 | 150089 | 150221 | *ycf2* |
| SD | palindromic | 20 | 150164 | 152449 | *ycf2* |

^*^ AU, *Aralia undulata*; BH, *Brassaiopsis hainla*; KS, *Kalopanax septemlobus*; MD, *Metapanax delavayi*; SD, *Schefflera delavayi*.
