# Supplementary material for: Complete Sequencing of Five Araliaceae Chloroplast Genomes and the Phylogenetic Implications
Source: PLoS One. 2013 Oct 18;8(10):e78568. doi: 10.1371/journal.pone.0078568 (PMC3799623; doi:10.1371/journal.pone.0078568)
Supplement: Table S3 — Indels in exons of genes in the seven Araliaceae chloroplast genomes. (DOCX) [file pone.0078568.s004.docx]

**Table S3.** Indels in exons of genes in the seven Araliaceae chloroplast genomes.

| **Gene** | **Numbers of indels** |
| --- | --- |
| *accD* | 2 |
| *atpF* | 1 |
| *ccsA* | 3 |
| *clpP* | 1 |
| *matK* | 3 |
| *ndhF* | 3 |
| *ndhI* | 1 |
| *psbH* | 1 |
| *rpl22* | 2 |
| *rpl32* | 1 |
| *rpoA* | 1 |
| *rpoB* | 2 |
| *rpoC1* | 1 |
| *rpoC2* | 4 |
| *rps18* | 1 |
| *ycf1* | 28 |
| *ycf2* | 9 |
| *ycf4* | 1 |
