# Supplementary material for: Complete Sequencing of Five Araliaceae Chloroplast Genomes and the Phylogenetic Implications
Source: PLoS One. 2013 Oct 18;8(10):e78568. doi: 10.1371/journal.pone.0078568 (PMC3799623; doi:10.1371/journal.pone.0078568)
Supplement: Table S4 — Summary statistics for candidate regions in the chloroplast genomes of Araliaceae. (DOCX) [file pone.0078568.s005.docx]

**Table S4.** Summary statistics for candidate regions in the chloroplast genomes of Araliaceae.

| **Regions^*^** | **Aligned length (bp)** | **No. of variable sites** | **Percentage of variable sites** | **No. of PIS^**^** | **Percentage of PIS** | **Location** |
| --- | --- | --- | --- | --- | --- | --- |
| ***trnK(UUU)-rps16****^***^* | 936 | 75 | 8.01 | 9 | 0.96 | LSC |
| ***trnC(GCA)-petN****^***^* | 739 | 59 | 7.98 | 11 | 1.49 | LSC |
| ***psbK-psbI****^***^* | 421 | 31 | 7.36 | 3 | 0.71 | LSC |
| ***trnE(UUC)-trnT(GGU)*** *^***^* | 868 | 62 | 7.14 | 15 | 1.73 | LSC |
| ***ycf1*** | 5866 | 399 | 6.80 | 95 | 1.62 | SSC |
| ***rps16-trnQ(UUG)*** | 1881 | 125 | 6.65 | 15 | 0.80 | LSC |
| ***psaJ-rpl33****^***^* | 475 | 30 | 6.32 | 3 | 0.63 | LSC |
| ***ndhF-rpl32****^***^* | 1259 | 79 | 6.27 | 14 | 1.11 | SSC |
| ***trnT(UGU)-trnL(UAA)*** *^***^* | 842 | 52 | 6.18 | 7 | 0.83 | LSC |
| ***rps15-ycf1****^***^* | 391 | 24 | 6.14 | 10 | 2.56 | SSC |
| ***rpl32-trnL(UAG)*** | 841 | 50 | 5.95 | 11 | 1.31 | SSC |
| ***petB-intron****^***^* | 795 | 47 | 5.91 | 21 | 2.64 | LSC |
| ***trnS(GCU)-trnG(UCC)*** | 762 | 45 | 5.91 | 5 | 0.66 | LSC |
| ***ycf3-trnS(GGA)*** *^***^* | 885 | 52 | 5.88 | 11 | 1.24 | LSC |
| ***ndhG-ndhI****^***^* | 394 | 23 | 5.84 | 2 | 0.51 | SSC |
| ***psaI-ycf4****^***^* | 414 | 24 | 5.80 | 1 | 0.24 | LSC |
| ***trnH(GUG)-psbA*** | 428 | 24 | 5.61 | 3 | 0.70 | LSC |
| ***ndhC-trnV(UAC)*** | 1035 | 57 | 5.51 | 7 | 0.68 | LSC |
| ***trnL(UAA)-intron****^***^* | 514 | 28 | 5.45 | 3 | 0.58 | LSC |
| ***trnF(GAA)-ndhJ****^***^* | 393 | 21 | 5.34 | 5 | 1.27 | LSC |
| ***accD-psaI*** | 736 | 39 | 5.30 | 6 | 0.82 | LSC |
| ***atpF-atpH****^***^* | 397 | 21 | 5.29 | 10 | 2.52 | LSC |
| ***petN-psbM*** | 915 | 48 | 5.25 | 8 | 0.87 | LSC |
| ***psbE-petL*** | 1182 | 62 | 5.25 | 7 | 0.59 | LSC |
| ***petA-psbJ*** | 1071 | 56 | 5.23 | 9 | 0.84 | LSC |
| ***ndhA-intron****^***^* | 1114 | 56 | 5.03 | 14 | 1.26 | SSC |
| *trnT(GGU)-psbD* | 1432 | 69 | 4.82 | 8 | 0.56 | LSC |
| *trnG(UCC)-intron* | 714 | 34 | 4.76 | 5 | 0.70 | LSC |
| *atpH-atpI* | 1137 | 54 | 4.75 | 15 | 1.32 | LSC |
| *rpl16-intron* | 981 | 44 | 4.49 | 10 | 1.02 | LSC |
| *ndhF* | 2276 | 102 | 4.48 | 32 | 1.41 | SSC |
| *psbM-trnD(GUC)* | 1186 | 53 | 4.47 | 13 | 1.10 | LSC |
| *rpoB-trnC(GCA)* | 1302 | 57 | 4.38 | 10 | 0.77 | LSC |
| *trnL(UAA)-trnF(GAA)^****^* | 373 | 16 | 4.29 | 5 | 1.34 | LSC |
| *atpB-rbcL* | 797 | 34 | 4.27 | 5 | 0.63 | LSC |
| *clpP-intron* | 1447 | 59 | 4.08 | 21 | 1.45 | LSC |
| *rpl14* | 369 | 15 | 4.07 | 1 | 0.27 | LSC |
| *atpF-intron* | 734 | 29 | 3.95 | 8 | 1.09 | LSC |
| *clpP-psbB* | 459 | 18 | 3.92 | 1 | 0.22 | LSC |
| *rpl22* | 486 | 19 | 3.91 | 6 | 1.23 | LSC |
| *trnP(UGG)-psaJ* | 412 | 16 | 3.88 | 4 | 0.97 | LSC |
| *ccsA* | 966 | 35 | 3.62 | 5 | 0.52 | SSC |
| *rbcL-accD* | 691 | 25 | 3.62 | 6 | 0.87 | LSC |
| *rps16-intron* | 893 | 32 | 3.58 | 7 | 0.78 | LSC |
| *atpF* | 555 | 19 | 3.42 | 5 | 0.90 | LSC |
| *matK* | 1518 | 51 | 3.36 | 7 | 0.46 | LSC |
| *petD-intron* | 772 | 25 | 3.24 | 6 | 0.78 | LSC |
| *rpl20* | 387 | 12 | 3.10 | 2 | 0.52 | LSC |
| *psaA-ycf3* | 750 | 23 | 3.07 | 7 | 0.93 | LSC |
| *rpoC1-intron* | 789 | 24 | 3.04 | 4 | 0.51 | LSC |
| *rpoC2* | 4197 | 123 | 2.93 | 18 | 0.43 | LSC |
| *clpP* | 591 | 17 | 2.88 | 4 | 0.68 | LSC |
| *atpE* | 423 | 12 | 2.84 | 2 | 0.47 | LSC |
| *rpoA* | 1064 | 30 | 2.82 | 3 | 0.28 | LSC |
| *trnS(UGA)-psbZ* | 355 | 10 | 2.82 | 1 | 0.28 | LSC |
| *rbcL* | 1437 | 39 | 2.71 | 13 | 0.90 | LSC |
| *trnK(UUU)-intron* | 1033 | 28 | 2.71 | 5 | 0.48 | LSC |
| *ndhD* | 1503 | 39 | 2.59 | 4 | 0.27 | SSC |
| *ycf3-intron* | 1486 | 38 | 2.56 | 4 | 0.27 | LSC |
| *accD* | 1529 | 39 | 2.55 | 7 | 0.46 | LSC |
| *rps3* | 660 | 16 | 2.42 | 2 | 0.30 | LSC |
| *rpl20-rps12* | 784 | 19 | 2.42 | 3 | 0.38 | LSC |
| *rps11* | 417 | 10 | 2.40 | 2 | 0.48 | LSC |
| *ndhA* | 1092 | 26 | 2.38 | 3 | 0.27 | SSC |
| *trnV(UAC)-intron* | 592 | 14 | 2.36 | 4 | 0.68 | LSC |
| *rps8* | 405 | 9 | 2.22 | 2 | 0.49 | LSC |
| *rpl16* | 408 | 9 | 2.21 | 1 | 0.25 | LSC |
| *ycf4* | 555 | 11 | 1.98 | 3 | 0.54 | LSC |
| *rps2* | 711 | 14 | 1.97 | 1 | 0.14 | LSC |
| *ndhC* | 363 | 7 | 1.93 | 1 | 0.28 | LSC |
| *ndhJ* | 477 | 9 | 1.89 | 2 | 0.42 | LSC |
| *petA* | 963 | 18 | 1.87 | 7 | 0.73 | LSC |
| *rpoC1* | 2070 | 37 | 1.79 | 10 | 0.48 | LSC |
| *ndhI* | 504 | 9 | 1.79 | 3 | 0.60 | SSC |
| *ndhG* | 531 | 9 | 1.69 | 3 | 0.56 | SSC |
| *ndhK* | 678 | 11 | 1.62 | 2 | 0.29 | LSC |
| *atpI* | 744 | 12 | 1.61 | 4 | 0.54 | LSC |
| *atpA* | 1524 | 23 | 1.51 | 6 | 0.39 | LSC |
| *rpoB* | 3216 | 47 | 1.46 | 8 | 0.25 | LSC |
| *psbC* | 1422 | 20 | 1.41 | 4 | 0.28 | LSC |
| *psbB* | 1527 | 21 | 1.38 | 6 | 0.39 | LSC |
| *atpB* | 1497 | 20 | 1.34 | 3 | 0.20 | LSC |
| *cemA* | 690 | 9 | 1.30 | 1 | 0.14 | LSC |
| *psaB* | 2205 | 27 | 1.22 | 5 | 0.23 | LSC |
| *psaA* | 2253 | 25 | 1.11 | 7 | 0.31 | LSC |
| *psbD* | 1062 | 10 | 0.94 | 2 | 0.19 | LSC |
| *psbA* | 1062 | 6 | 0.56 | 1 | 0.09 | LSC |
| *petB* | 648 | 3 | 0.46 | 1 | 0.15 | LSC |

^*^ Fragments rank from most variable to least variable. The top 26 fragments are indicated in boldface.

^**^ PIS: parsimony-informative sites.

^***^ Regions are newly identified in our current study.

^****^ Fragments have been used in previous phylogenetic studies in Araliaceae.
